# Pervasive hybridization during evolutionary radiation of *Rhododendron* subgenus *Hymenanthes* in mountains of southwest China

**DOI:** 10.1093/nsr/nwac276

**Published:** 2022-12-02

**Authors:** Yazhen Ma, Xingxing Mao, Ji Wang, Lei Zhang, Yuanzhong Jiang, Yuying Geng, Tao Ma, Liming Cai, Shuangquan Huang, Pete Hollingsworth, Kangshan Mao, Minghui Kang, Yiling Li, Wenlu Yang, Haolin Wu, Yang Chen, Charles C Davis, Nawal Shrestha, Richard H Ree, Zhenxiang Xi, Quanjun Hu, Richard I Milne, Jianquan Liu

**Affiliations:** Key Laboratory for Bio-Resource and Eco-Environment of Ministry of Education, College of Life Sciences, Sichuan University, Chengdu 610065, China; State Key Laboratory of Grassland Agro-ecosystem, College of Ecology, Lanzhou University, Lanzhou 730000, China; Key Laboratory for Bio-Resource and Eco-Environment of Ministry of Education, College of Life Sciences, Sichuan University, Chengdu 610065, China; Key Laboratory for Bio-Resource and Eco-Environment of Ministry of Education, College of Life Sciences, Sichuan University, Chengdu 610065, China; Key Laboratory for Bio-Resource and Eco-Environment of Ministry of Education, College of Life Sciences, Sichuan University, Chengdu 610065, China; Key Laboratory for Bio-Resource and Eco-Environment of Ministry of Education, College of Life Sciences, Sichuan University, Chengdu 610065, China; Key Laboratory for Bio-Resource and Eco-Environment of Ministry of Education, College of Life Sciences, Sichuan University, Chengdu 610065, China; Key Laboratory for Bio-Resource and Eco-Environment of Ministry of Education, College of Life Sciences, Sichuan University, Chengdu 610065, China; Department of Organismic and Evolutionary Biology and Harvard University Herbaria, Harvard University, Cambridge, MA 02138, USA; Institute of Evolution and Ecology, School of Life Sciences, Central China Normal University, Wuhan 430079, China; Royal Botanic Garden Edinburgh, Edinburgh EH3 5LR, UK; Key Laboratory for Bio-Resource and Eco-Environment of Ministry of Education, College of Life Sciences, Sichuan University, Chengdu 610065, China; Key Laboratory for Bio-Resource and Eco-Environment of Ministry of Education, College of Life Sciences, Sichuan University, Chengdu 610065, China; Key Laboratory for Bio-Resource and Eco-Environment of Ministry of Education, College of Life Sciences, Sichuan University, Chengdu 610065, China; Key Laboratory for Bio-Resource and Eco-Environment of Ministry of Education, College of Life Sciences, Sichuan University, Chengdu 610065, China; Key Laboratory for Bio-Resource and Eco-Environment of Ministry of Education, College of Life Sciences, Sichuan University, Chengdu 610065, China; Key Laboratory for Bio-Resource and Eco-Environment of Ministry of Education, College of Life Sciences, Sichuan University, Chengdu 610065, China; Department of Organismic and Evolutionary Biology and Harvard University Herbaria, Harvard University, Cambridge, MA 02138, USA; State Key Laboratory of Grassland Agro-ecosystem, College of Ecology, Lanzhou University, Lanzhou 730000, China; Negaunee Integrative Research Center, Field Museum, Chicago, IL 60605, USA; Key Laboratory for Bio-Resource and Eco-Environment of Ministry of Education, College of Life Sciences, Sichuan University, Chengdu 610065, China; Key Laboratory for Bio-Resource and Eco-Environment of Ministry of Education, College of Life Sciences, Sichuan University, Chengdu 610065, China; Royal Botanic Garden Edinburgh, Edinburgh EH3 5LR, UK; Institute of Molecular Plant Sciences, The University of Edinburgh, Edinburgh EH9 3JH, UK; Key Laboratory for Bio-Resource and Eco-Environment of Ministry of Education, College of Life Sciences, Sichuan University, Chengdu 610065, China; State Key Laboratory of Grassland Agro-ecosystem, College of Ecology, Lanzhou University, Lanzhou 730000, China

**Keywords:** evolutionary radiation, hybridization, rhododendron, subgenus *Hymenanthes*, montane flora

## Abstract

Radiations are especially important for generating species biodiversity in mountainous ecosystems. The contribution of hybridization to such radiations has rarely been examined. Here, we use extensive genomic data to test whether hybridization was involved in evolutionary radiation within *Rhododendron* subgenus *Hymenanthes*, whose members show strong geographic isolation in the mountains of southwest China. We sequenced genomes for 143 species of this subgenus and 93 species of four other subgenera, and found that *Hymenanthes* was monophyletic and radiated during the late Oligocene to middle Miocene. Widespread hybridization events were inferred within and between the identified clades and subclades. This suggests that hybridization occurred both early and late during diversification of subgenus *Hymenanthes*, although the extent to which hybridization, speciation through mixing-isolation-mixing or hybrid speciation, accelerated the diversification needs further exploration. Cycles of isolation and contact in such and other montane ecosystems may have together promoted species radiation through hybridization between diverging populations and species. Similar radiation processes may apply to other montane floras in this region and elsewhere.

## INTRODUCTION

A central question in evolution concerns the mechanisms underlying rapid radiation, i.e. the generation of large numbers of morphologically diverse species from a single lineage over a short evolutionary time span, and often within a relatively narrow geographical range [1,2]. Such radiations are especially common in mountainous regions, oceanic islands and rift lakes [3–8]. Genomic admixtures caused by hybridization have repeatedly been found to drive radiations in the latter two ecosystems [9–11]. However, although hybridizaion has been reported in many continental plant groups [12–14], this possibility has not been fully explored based on genomic data in mountainous systems, and even less so in alpine plant radiations. Ecological and geographical isolation are assumed to drive species radiations in mountainous regions [15,16] because tectonic uplift and accompanying environmental changes generate high mountains alternating with deep valleys that provide more available niches [17]. Such mountains can form so-called ‘sky islands’ in which climatic oscillations can cause cycles of connection and isolation between populations or species occurring there, which may result in hybridization [18,19]. Moreover, recent modelling of speciation shows that such mixing-isolation-mixing (MIM) cycles could greatly accelerate species diversification [20]. Hence, the role of hybridization as a contributor to other major plant radiations requires investigation, especially in mountainous ecosystems, where there are strong effects from ecological and geographic isolation, plus frequent hybridization events [21].

Testing the role of hybridization in plant radiations remains challenging because genomic admixture can result from any of four evolutionary outcomes after hybridization, depending on timescales. First, hybrid zones may form containing many hybrid derivatives, but without necessarily altering the parent species or leading to speciation [22]. Second, backcrossing might lead to germplasm transfer through introgression, altering the recipient species either locally or, ultimately, throughout its range [22], but without causing speciation. Third, hybridization may occur between diverging populations through natural selection and geographic isolation and such divergence with gene flow can promote speciation [20]. Finally, hybrid species may arise between two already well-differentiated species [23] and this new lineage can then generate more species through subsequent speciation events [24]. Only in the latter two scenarios, where it triggered or promoted the origin of extant species or clades, can hybridization be said to have contributed significantly to evolutionary radiation. However, the signatures of such events can be hard to distinguish from recent hybridization events that did not generate new species, such as recent introgression, especially if only a small number of individuals are sampled. This is a particular issue in groups where hybridization and introgression are very common, even between morphologically and phylogenetically distant species [25].

*Rhododendron* subgenus *Hymenanthes* is an iconic example of plant radiation, containing around 302 species, most of them diploid and highly interfertile, with the great majority occurring in the mountains of southwest China, where many are endemic to single mountains [26–29]. All species of this subgenus are outcrossing, with extreme diversity in flowers, pollinators and ecological preference [30,31]. Small and light rhododendron seeds are mainly dispersed by wind and animals [32] giving the potential to reach sites relatively distant from the parent plant [33]. As found for other groups with evolutionary radiation [34], species delimitation in this subgenus is challenging due to morphological variation within species, and even populations [35]. All previous phylogenetic studies indicate that *Hymenanthes* species comprise a monophyletic group [36–38], but within the group, discordance between cpDNA, nuclear DNA and morphology appear to indicate ancient hybridization events [36–39]. In addition, numerous natural hybrids have also been reported [40,41], so there is great potential for recent interspecific gene flow as well. In this study, we aimed to examine the contribution of historical hybridization to the evolutionary radiation of *Hymenanthes*. We assembled a high-quality genome for one *Hymenanthes* species (*Rhododendron prattii*). We then performed whole-genome sequencing for 164 individuals of 143 species through selecting typical samples after genotyping available populations for the widespread species of this subgenus. Next, we constructed a dated phylogeny of the subgenus, and used this to seek signals of hybridization within and between clades, subclades and species. We uncovered extensive hybridization, both early and late during the diversification of subgenus *Hymenanthes*.

## RESULTS

### Reference genome assembly and annotation

We generated a chromosome level genome assembly for one member of subgenus *Hymenanthes, Rhododendron prattii*, by integrating single-molecule real-time (SMRT) sequencing, Illumina sequencing, 10x Genomics and high-throughput chromosome conformation capture (Hi-C mapping) techniques (Table S1). This *de novo* assembly was 673 Mb in size with 631 Mb sequences anchored onto 13 chromosomes and scaffolds N50 of 47.1 Mb (Figs S1 and S2, and Tables S2 and S3). Benchmarking universal single-copy orthologs (BUSCO) analysis indicated that the assembled genome obtained 97.3% of the embryophyta universal single-copy orthologs, confirming the high completeness of the assembly; 57.26% of the genome sequences were identified as repetitive elements. Among them, long terminal repeat (LTR) retrotransposons were the most abundant, accounting for 32.14% of the genome (Table S4). A total of 37 092 protein-coding genes were predicted for the assembly and 36 523 (96.1%) could be annotated by at least one public database (Tables S5 and S6). The repeat content and predicted gene number are similar to those of the other five reported species in genus *Rhododendon* (Table S7). All annotated genes in these six high-quality *Rhododendron* genomes could be assigned to 26 372 gene families, which comprise 5415 single-copy orthologous genes across them in the total genus.

### Calibrated-phylogeny and recent radiation of subgenus *hymenanthes*

To ensure a sufficient species sampling for the radiation analyses, we examined around 12 000 individuals comprising ca. 800 populations of 292 species of subgenus *Hymenanthes* distributed in southwest China and other regions [28]. If a species had clear morphological gaps from other species, it was selected for further investigation. To rule out recent cryptic introgression, we genotyped widespread species that had more than four examined populations using 15 pairs of nuclear simple sequence repeat (SSR) markers [25] and chose one to three individuals that represent a relatively pure genetic composition of each species for final genome sequencing (see Supplementary Materials and Methods; Fig. S3). In total, we sequenced genomes of 164 individuals of 143 species (including subspecies and variety) for subgenus *Hymenanthes*, with a mean sequencing depth of 38× per individual (Table S8). In addition, 113 individuals of 93 species from four other *Rhododendron* subgenera were also selected for genome sequencing by examining wild populations in the same way (Table S8). By aligning all sequencing data to the *R. prattii* reference genome, we inferred the ploidy for each individual and found that all genome sequenced samples within *Hymenanthes* are diploid. The polyploid species detected in other subgenera were excluded from subsequent analyses (Table S9). After variants calling and stringent quality filtering, 14.8 million high-quality biallelic SNPs were obtained for the whole genus. The average sequence divergences (*d_XY_*) between subgenus *Hymenanthes* and other subgenera ranged from 0.035 to 0.048 (Fig. S4) and that between species within subgenus *Hymenanthes* was 0.018 (Fig. S5). We found that sequence diversities within individuals (heterozygosity) of *R. agastum* (0.022), *R. catawbiense* (0.026) and *R. adenopodum* (0.026) were obviously higher than those of the other sampled species—from 0.005 to 0.018 per base pair. Therefore, these three ‘species’ may comprise F1s as suggested before [41] and were further excluded in the following analyses (see Supplementary Materials and Methods).

**Figure 1.**
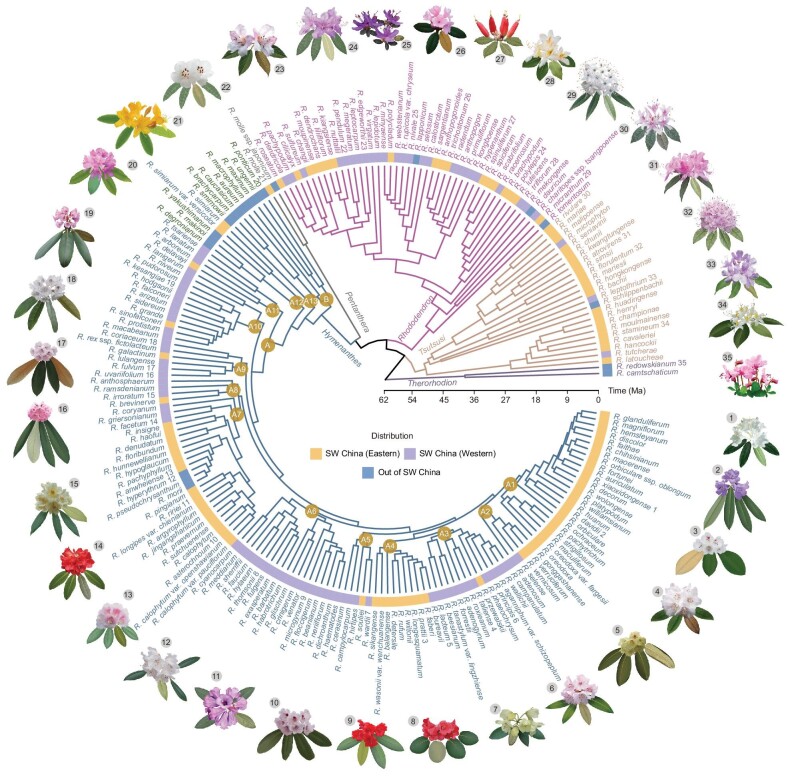
Time-calibrated species tree of subgenus *Hymenanthes*. The species tree was calibrated based on a maximum-likelihood topology inferred from whole-genome SNPs. Branches are coloured according to different subgenera and those marked in blue belong to subgenus *Hymenanthes*. The outer circle represents the distribution of each species. Names of each clade and subclade are indicated in the ocher circles. The outer illustrations are 35 representative species from the tree for which the illustration IDs are marked behind species names. Names of *Hymenanthes* species from Tertiary relict regions are marked in green. SW China, southwest China and adjacent regions.

Principal component analysis (PCA) of genome-wide polymorphisms distinctly separated subgenus *Hymenanthes* from the four other subgenera by the first two PCs (Fig. S6). We inferred phylogenetic relationships among all sampled species based on three datasets: all nuclear genome SNPs (6 448 516), SNPs from protein-coding regions of the 4 468 single-copy nuclear genes only (483 944), and all SNPs from the chloroplast genome (12 273). Both concatenation and coalescent analyses supported the monophyly of subgenus *Hymenanthes* with a sister relationship to subgenus *Pentanthera* (Fig. 1 and Figs S7–S15). We used the concatenated nuclear genome tree to date the crown divergence and diversification dynamics within subgenus *Hymenanthes*, using the ∼56 Ma (million years ago) old fossil of *Rhododendron newburyanum* for calibration [42]. We found that the basal clade within *Hymenanthes* diverged from others at about 35.4 Ma (95% confidence interval: 31.5 to 41.3 Ma; Fig. 1). Since then, subgenus *Hymenanthes* showed increased diversification rates from the late Oligocene to middle Miocene (Fig. S16). This acceleration of diversification occurred during a time of increased geological and climatic oscillations in southwest China [21].

### Phylogenetic discordance and shared polymorphisms

**Figure 2.**
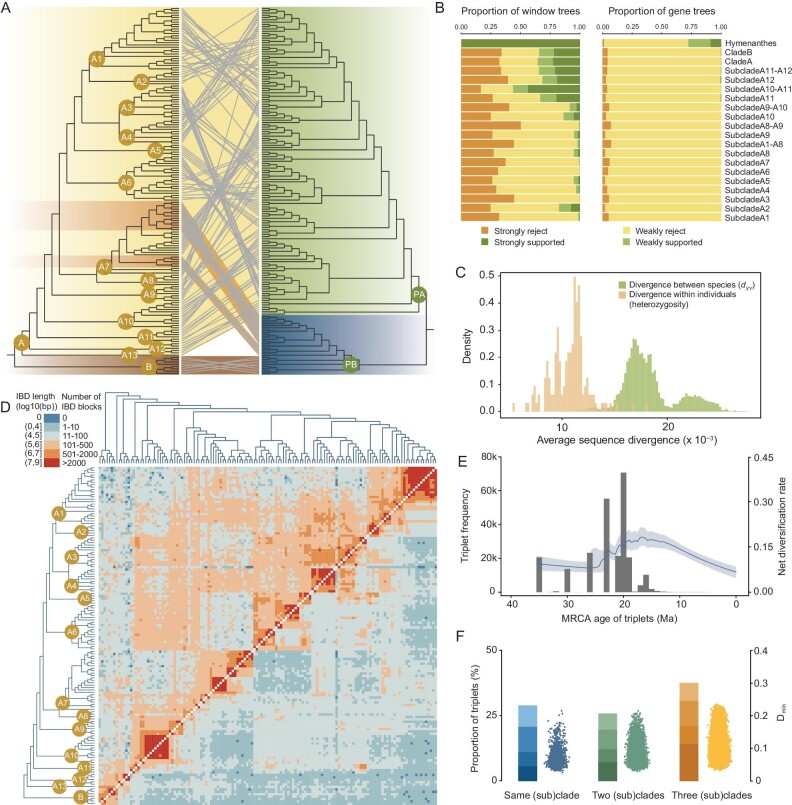
Topological conflicts and extensive hybridization during evolutionary diversification of subgenus *Hymenanthes*. (A) Comparison of a maximum-likelihood phylogeny inferred from whole-genome SNPs (left) and a ML phylogeny based on the plastome SNPs (right). Eleven species within subclade A7 descended from an ancient hybridization event between the two initial diverging lineages of *Hymenanthes* are highlighted with orange background. (B) Local 10k-SNP window tree and gene tree compatibility revealed by the portion of trees for which clades (*y*-axis) are highly (weakly) supported or rejected. Weakly rejected clades are those not in the tree but being compatible if low support branches (<75%) are contracted. (C) Distributions of genomic divergence between species (*d_XY_*) and sequence diversity within individuals (heterozygosity) for subgenus *Hymenanthes*. (D) Estimated haplotype sharing in all sampled species within subgenus *Hymenanthes*. Heatmap colors represent the total length (above the diagonal) and the total number (below the diagonal) of identity-by-descent blocks for each species pairwise comparison. (E) Timescale distribution of the most recent common ancestor (MRCA) ages for the triplets with significantly elevated *D* values resulting from hybridization against the dynamic of net diversification rate of *Hymenanthes*. Ma, million years ago. (F) Excess allele sharing suggests non-bifurcating relationships among triplets of species within subgenus *Hymenanthes*. The bars denote the proportion of significantly elevated *D*_min_ values with the shading representing family-wise error rate (FWER, see Supplementary Materials and Methods) of 5 × 10^−2^, 5 × 10^−4^, 5 × 10^−8^ and 5 × 10^−14^ (from light to dark). The scatterplots represent the *D*_min_ scores that were significantly different from zero with FWER < 5 × 10^−2^. Results are shown separately for cases where the three members of a triplet come from the same clade (or subclade), two clades (or subclades), or three separate clades (or subclades).

Relationships among species within subgenus *Hymenanthes* were largely incongruent between phylogenies inferred from different methods or datasets, especially for species from southwest China (Figs 1 and 2A, and Figs S7–S15). Based on the nuclear genome ML tree, two clades were identified within subgenus *Hymenanthes*. Clade A comprised all sampled species from southwest China and surrounding regions along with four species from Japan and southwestern Eurasia which occupied basal positions, while clade B was formed by seven species distributed in North America, southwestern Eurasia and northeastern Asia. Thirteen subclades were further recovered within clade A (Fig. 1). The plastome topology, however, differs markedly from the phylogenetic trees obtained with nuclear genome, although all sampled species are again divided into two clades (PA and PB; Fig. 2A). The clade PA contained the same four species from southwestern Eurasia and northeastern Asia in basal positions as did clade A, plus a huge monophyletic subclade comprising all but 11 of the sampled species from southwest China and adjacent regions. The remaining 11 species from southwest China and neighboring areas formed a monophyletic subclade within clade PB, nested among the seven members of nuclear clade B, from other parts of the Northern Hemisphere (Figs 1 and 2A and Fig. S15). In addition, topological conflicts among local SNP window trees and gene trees were also widely observed among species within the subgenus (Fig. S17). In the nuclear genome ML tree, we found high levels of discordance for window trees as well as gene trees in most focal splits among major clades/subclades, except for the ancestral node of the subgenus (Fig. 2B and Fig. S18). However, for all of the focal splits, the majority of the gene/window trees are compatible with the species tree after contracting those branches with low support (below 75%).

Consistent with the expectations of reticulate evolution, we observed extensive shared polymorphisms among species. The average sequence divergences between species (*d_XY_*) within this subgenus ranged from 0.009 to 0.028, and partially overlapped with the distribution of sequence diversity within individuals (heterozygosity; Fig. 2C). In certain cases, sequence divergence within an individual is higher than divergence between species (Fig. 2C), suggesting that some of the species produced during radiation are genetically very closely related. The *d_XY_* values follow a bimodal distribution. The first peak mainly indicates the sequence differences between species distributed in southwest China, i.e. all of clade A except for the two basal subclades (A12 and A13). The second peak is composed of *d_XY_* values between species from southwest China and those with a Tertiary relict distribution (species from subclades A12 and A13, and clade B), and between species within the Tertiary relict group (Fig. S5). The nucleotide diversities of each clade and subclade ranged from 0.007 to 0.020 and no correlation was found between nucleotide diversity and the number of species in each clade/subclade. We then explored the identical-by-descent (IBD) haplotypes, of which 2 480 269 were detected within subgenus *Hymenanthes* (Fig. 2D). As expected, extensive sharing of haplotypes between species was detected, showing evidence of recent interspecific gene flow, for example between *R. delavayi* and *R. irroratum* (number of IBD = 140, total IBD length = 1.05 Mb, maximum IBD length = 135.75 kb), and between *R. decorum* and *R. vernicosum* (number of IBD = 299, total IBD length = 2.08 Mb, maximum IBD length = 121.02 kb). Furthermore, excess haplotype sharing was widely recovered between species from distinct subclades with overlapping distributions. For example, there was considerable haplotype sharing between subclades A1 and A7 in the eastern part of southwest China, and strong signatures of genetic admixture between subclades A6 and A10 in the Hengduan Mountains and the Himalayan region (Fig. 2D).

### Tests of historical hybridization events during evolutionary radiation

To further determine the historical role of hybridization in causing these shared polymorphisms and gene tree discordance, we calculated Patterson's *D* statistics (the ABBA-BABA test) for every possible triplet among the sampled species. The lowest absolute value of the *D*-statistic (*D*_min_) across all possible tree topologies was determined for each triplet. In 153 186 of 447 581 triplets (34.2%) within subgenus *Hymenanthes, D*_min_ values differed significantly from zero, indicating hybridization among the triplets. Furthermore, most triplets with an elevated *D* score had common ancestors dated mainly from the late Oligocene to middle Miocene, when diversification within *Hymenanthes* was starting to accelerate (Fig. 2E and Fig. S16). Based on these results, we also calculated the Reticulation Index for each node in the inferred species tree, to quantify the intensity of gene flow at each node. We found that the reticulation indices were high throughout the whole phylogeny, especially for lineages distributed in southwest China, suggesting ancient and persistent hybridization contributed substantially to the radiation of this subgenus (Fig. S19). Furthermore, widespread excess allele sharing between species was detected, both within and across major lineages, clades and subclades, revealing reticulate evolution at multiple levels (Fig. 2F).

**Figure 3.**
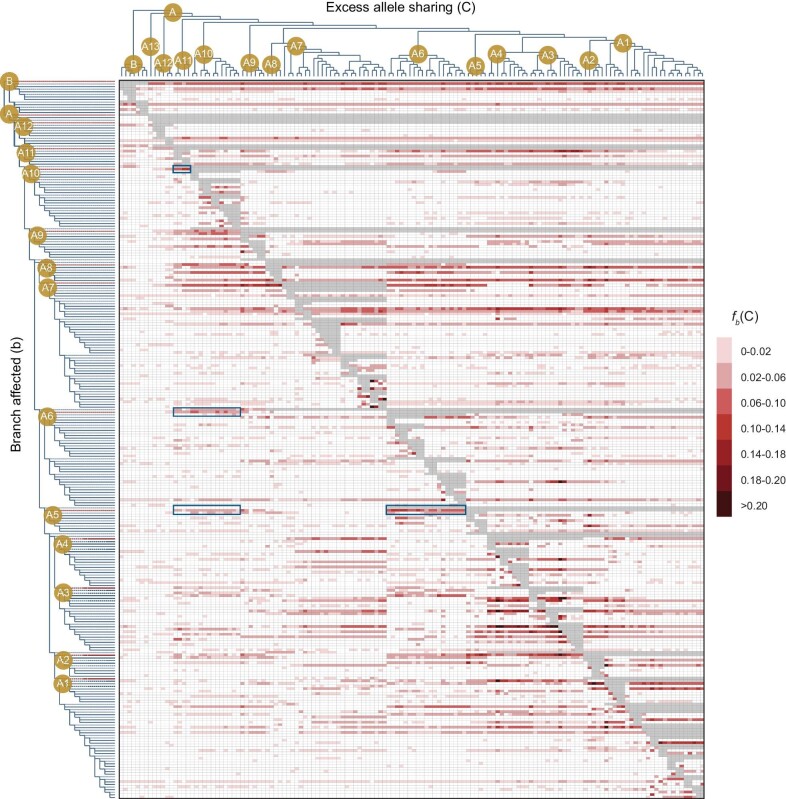
Quantification of gene flow between branches in the species tree of subgenus *Hymenanthes*. The branch-specific statistic *f_b_*(*C*) value shows excess sharing of derived alleles between a given branch (b) on the *y*-axis, relative to its sister branch, and the species C on the *x*-axis. The ML tree based on whole-genome SNPs was used as a basic topology for the branch statistic. Branches marked in red on the *y*-axis represent the ancestral branch for each clade or subclade. Blue rectangles indicate four cases of significant signals of excess allele sharing, between the common ancestors of (i) subclade A10 and species from subclade A11, (ii) subclade A6 and species from subclades A10 and A11, (iii) subclade A5 and species from subclades A10 and A11, and (iv) subclade A5 and species from subclade A6.

We then examined whether such multi-level hybridization events occurred between different branches of the species tree using the ‘*f*-branch’ metric. This method detects excess allele sharing between a species *C* and a branch *b* when compared to the branch that is sister to *b* on a given species tree. In total, 22.7% (8 042 out of 35 316) of species-branch pairs had significantly elevated *f_b_*(*C*) scores (a summary of *f*_4_ admixture ratios; see Supplementary Materials and Methods; Fig. 3), and a total of 238 of 278 branches in the nuclear species tree showed significant excess allele sharing with at least one other species. Among these, 109 ancestral branches of multiple species showed significant elevated *f_b_*(*C*) scores, again suggesting that hybridization events frequently occurred among ancestral lineages of clades or subclades, and moreover that in some cases one single ancestral hybridization event may have been involved in the origins of multiple daughter species. Many signals of excess allele sharing for a specific branch are hierarchical, suggesting repeated hybridization events between different clades/subclades during radiation.

## DISCUSSION

Hybridization has long been assumed to promote species radiation, and this has been well studied in animals in the oceanic islands and rift lakes [9–11], but rarely tested within the species-rich clades that occur in mountainous ecosystems. In this study, we used whole-genome resequencing data to examine historical hybridization events underlying evolutionary radiation within *Rhododendron* subgenus *Hymenanthes*, which has high species diversity, especially in the mountains of southwest China. We found extensive hybridization and genomic admixture during the historical radiation of this subgenus, and for the first time revealed that repeated isolation and hybridization may have promoted rapid species diversification in mountainous regions. Hybridization can lead to various outcomes, including introgression of material between species and possible homogenization, but also promotion of species diversification through gene flow, and the origin of new lineages via homoploid or polyploid hybrid speciation [20,22–24]. The signature of ancient hybridization promoting such species radiations can be masked by more recent hybridization that leads to hybrid zones, and introgression within and between extant species [24]. Therefore, we genotyped multiple natural populations of the widespread species, in order to exclude possible recent introgression and hybrids, and from this we selected individuals for whole-genome sequencing and final analyses. This minimized the confounding effects of recent introgression, aiding the detection of more ancient hybridization that contributed to species diversification.

Although variant calling bias cannot be completely avoided by mapping the genome-sequencing data from other subgenera to one reference genome from subgenus *Hymenanthes*, our phylogenetic analyses of three different genome-scale datasets obtained the same relationships among different subgenera as previous studies, and supported the monophyly of subgenus *Hymenanthes* as found previously [36–38]. In particular, with genome-wide sampling across the nuclear and plastid genomes and a greater taxon coverage than any previous study of the subgenus, we obtained the most detailed phylogenetic relationships between lineages and species within the subgenus so far (Fig. 1 and Figs S7–S15). Our results strongly rejected most taxonomic treatments that were based on morphological traits [27] and likewise most subsections were not monophyletic. For example, subsections *Fortunea* and *Argyrophylla*, were highly polyphyletic with the sampled species of each placed in different clades/subclades. We also recovered extensive topological conflicts between phylogenies based on different datasets, especially between nuclear genome and plastome trees (Fig. 2A and B), which indicated that multiple hybridization events might have occurred during the diversification of this subgenus. For example, our nuclear phylogeny revealed a clade of seven Tertiary relict species (clade B in Fig. 1; distributed in North America, southwestern Eurasia, and northeastern Asia), which was not present in previous nuclear phylogenies based on single nuclear regions [36,37]. Similar to a previous cpDNA phylogeny [39], a clade containing the same seven species was also basal in the plastome phylogeny, but here it included a subclade of 11 sino-Himalayan species nested within it, indicating that these 11 species descended from a hybridization event between the two initial diverging lineages of *Hymenanthes*, probably around central Sichuan where most of these species occur. These 11 species occupy a derived position in the nuclear phylogeny, so the event occurred relatively late during the diversification of *Hymenanthes*. Furthermore, another 11 species with clade PA plastome types are nested among them in the nuclear phylogeny, indicating several other hybridization events in this clade later on. Any such event might have been hybrid speciation or introgression of nuclear germplasm and/or plastids; however, the initial event certainly represents contact between lineages with markedly different geographical ranges. In addition, extensive IBD haplotype sharing across species and significant signals of excess allele sharing between species or lineages indicated by ABBA-BABA tests further confirmed that hybridization events had occurred between clades, subclades and species, and hence to have occurred at different times throughout the diversification of *Hymenanthes* (Figs 2 and 3). However, it should be noted that as instances of historical hybridization are so pervasive, the signal of the oldest such events might be obscured by slightly younger events.

**Figure 4.**
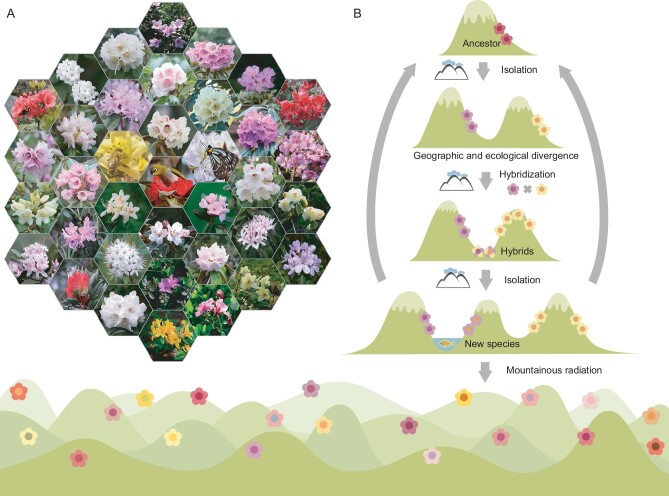
Simplified schematic of radiative diversification of subgenus *Hymenanthes* in the mountains of southwest China. (A) Diverse flowers and pollinators of *Hymenanthes* and other subgenera. (B) Cycles of isolation and hybridization that drive radiative diversification and produce many morphologically similar species.

In addition to extensive hybridization and genomic admixture at deep and shallow timescales, our results also reveal that this subgenus radiated mainly from the late Oligocene to middle Miocene (Fig. S16), a period of significant geological and climatological change in southwest China [43]. These events might have promoted the accumulation of specific mutations by applying novel selection pressures, and new ecological niches that are regularly generated in such montane ecosystems [21]. However, climatic oscillations might have caused range expansions of species that had been previously isolated, leading to hybridization events, and hence the potential for alteration of lineages by introgression, and the formation of hybrid lineages [21]. Additionally, dispersing seeds across geographical barriers such as mountain chains or deep valleys [6] might have promoted hybridization and possible speciation. Rhododendrons are outcrossing but employ a diverse range of pollinators for sexual reproduction and seed set (Fig. 4A) [44–46]. Different birds and insects were observed to visit the same species, but birds seem to be more effective pollinators than insects for large-flowered species at high-altitudes where the temperature is low [44]. If birds travel long distances and are not faithful to particular species, these traits might promote gene flow via pollination between populations or species occurring on the isolated mountains. Equally, ecological and geographic isolation between populations, and variation between their niches, might have promoted further divergence among isolated lineages, including those generated or altered by hybridization. Such cycles of isolation and hybridization (Fig. 4B) might thus have created a positive feedback loop that generated many morphologically similar species.

Speciation with gene flow, i.e. hybridization between diverging populations before the formation of reproductive isolation, can accelerate speciation based on MIM modeling results [20], and hybrid speciation between already genetically diverged species without complete reproductive isolation can likewise contribute to the accumulation of species diversity [22–24]. A major question that remains is the extent to which hybridization accelerated the radiative diversification of *Hymenanthes*, and how many reticulation events could be discerned among all current lineages. Homoploid hybrid speciation is favored by the geographic and ecological isolation that is readily attained in montane ecosystems like the Himalaya and Hengduan Mountains of southwest China [47,48], but to prove it occurred within *Hymenanthes*, it must be shown that hybridization events contributed to reproductive isolation in addition to the genomic admixture revealed here [23,24]. It should be noted that cycles of isolation and hybridization may have been common throughout the world in mountainous regions that experienced similar geological dynamics and climatic oscillations [5]. Indeed, hybridization was reported to have frequently occurred in many more plant and animal species-rich genera in southwest China [21], and also the European Alps [49], the Northern American Rockies and Sierras [50], the Southern American Andes [51] and other montane ecosystems [17]. Therefore, such a model of evolutionary radiation involving isolation and hybridization is likely to apply not just to *Hymenanthes* in southwest China, but to other mountainous floras and even faunas worldwide.

## METHODS AND MATERIALS

Detailed descriptions of methods are available as Supplementary data.

## DATA AVAILABILITY

Whole-genome sequencing data, and transcriptome data have been deposited the Genome Sequence Archive in the National Genomics Data Center (https://bigd.big.ac.cn/) with accession number CRA005762. Genome assembly and annotation have been deposited in the Genome Warehouse under accession number GWHBHLU00000000.

## Supplementary Material

nwac276_Supplemental_Files
