# Supplementary material for: Pervasive hybridization during evolutionary radiation of Rhododendron subgenus Hymenanthes in mountains of southwest China
Source: Natl Sci Rev. 2022 Dec 2;9(12):nwac276. doi: 10.1093/nsr/nwac276 (PMC9844246; doi:10.1093/nsr/nwac276)
Supplement: nwac276_Supplemental_Files [file nwac276_supplemental_files.zip › Supplementary_Data-Figures.docx]

**P****ervasive hybridization during evolutionary radiation of *Rhododendron* subgenus *Hymenanthes* in mountains of southwest China**

Yazhen Ma^1,2^†, Xingxing Mao^1^†, Ji Wang^1^†, Lei Zhang^1^, Yuanzhong Jiang^1^, Yuying Geng^1^, Tao Ma^1^, Liming Cai^3^, Shuangquan Huang^4^, Pete Hollingsworth^5^, Kangshan Mao^1^, Minghui Kang^1^, Yiling Li^1^, Wenlu Yang^1^, Haolin Wu^1^, Yang Chen^1^, Charles C. Davis^3^,Nawal Shrestha^2^, Richard H. Ree^6^, Zhenxiang Xi^1^, Quanjun Hu^1^*, Richard I. Milne^5,7^*, Jianquan Liu^1,2^*

*Corresponding authors. Email: liujq@nwipb.cas.cn; r.milne@ed.ac.uk; huquanjun@scu.edu.cn.

Figs. S1 to S20


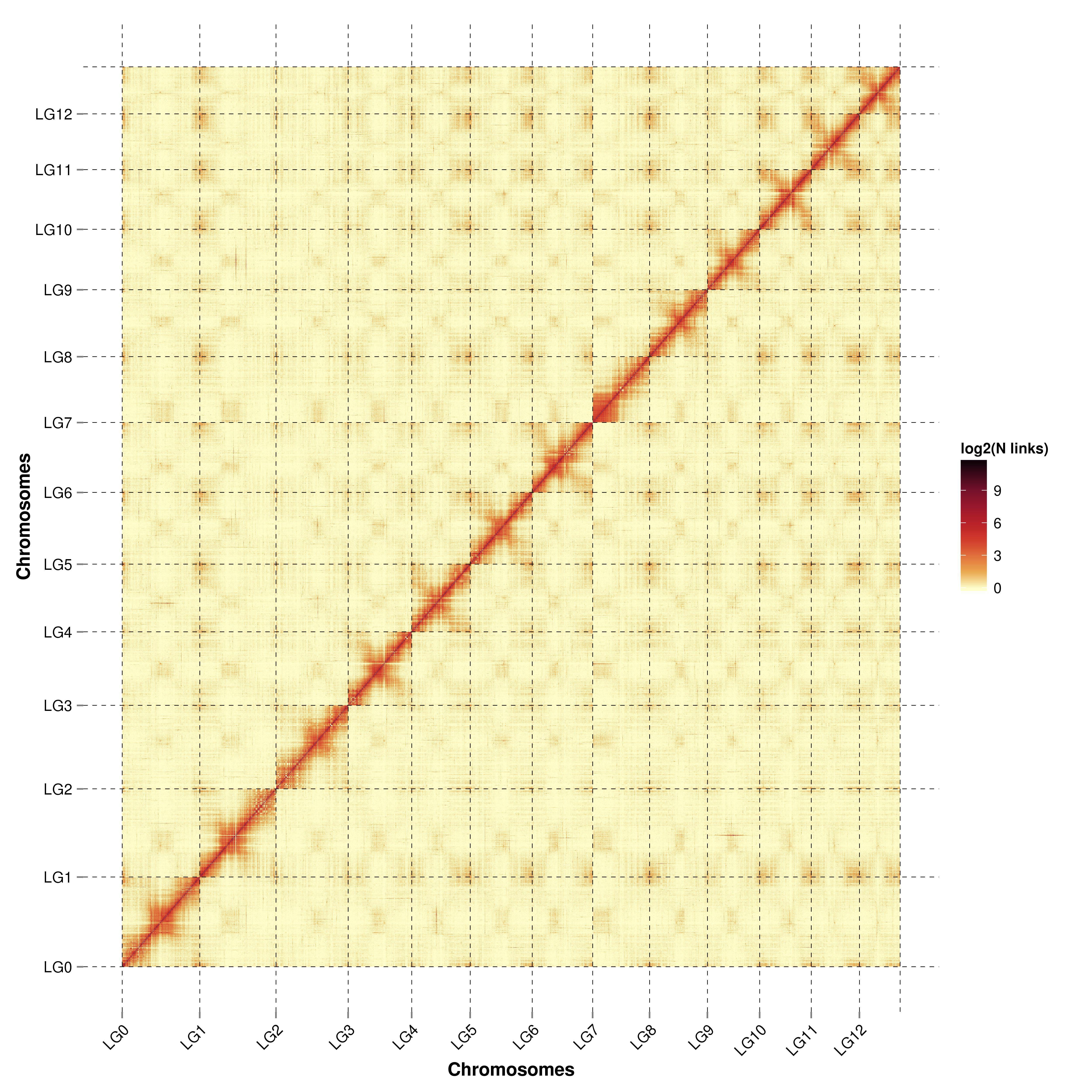


# Fig. S1. Interaction heat map of Hi-C links among chromosome groups for *R. prattii* from subgenus *Hymenanthes*. The assembled genomes were divided into 100-kb non-overlapping bins, and count of valid interaction links were calculated between each pair of bins. Frequency of Hi-C interaction links (displayed in the logarithm scale) is indicated by colours ranging from light yellow to dark red. LG0-LG12 represent the 13 chromosome groups inferred by LACHESIS.

**
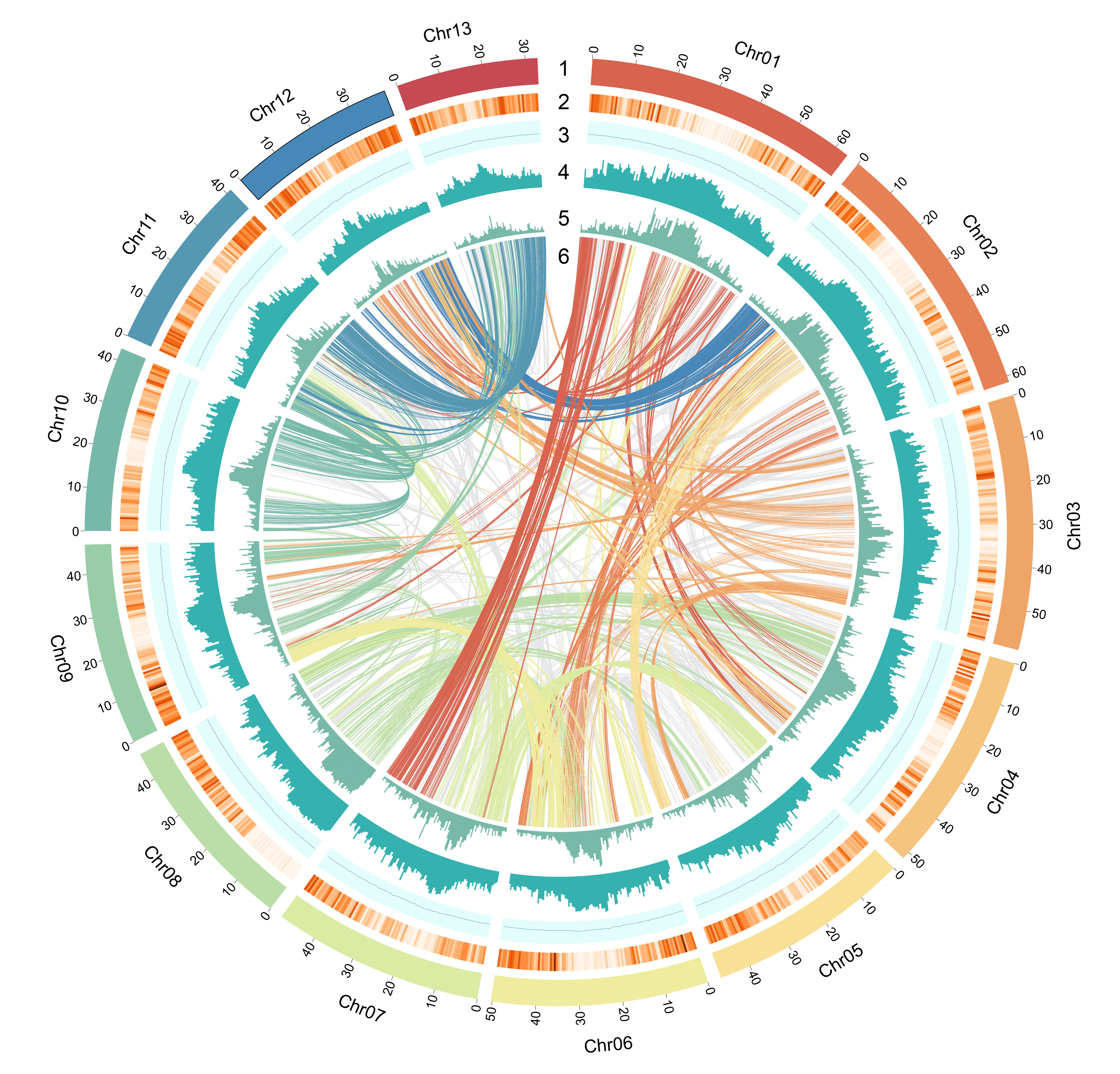
**

# Fig. S2. Genomic features of *R. prattii* from subgenus *Hymenanthes*. Tracks from outside to inside: 1, positions (in Mb) of the thirteen chromosomes; 2, gene density heat map (darker shade indicates higher density) over 500-kb chromosomal interval; 3, GC content; 4, repeat frequency; 5, long terminal repeat (LTR) retrotransposons frequency; 6, intragenomic syntenic relationships.

**
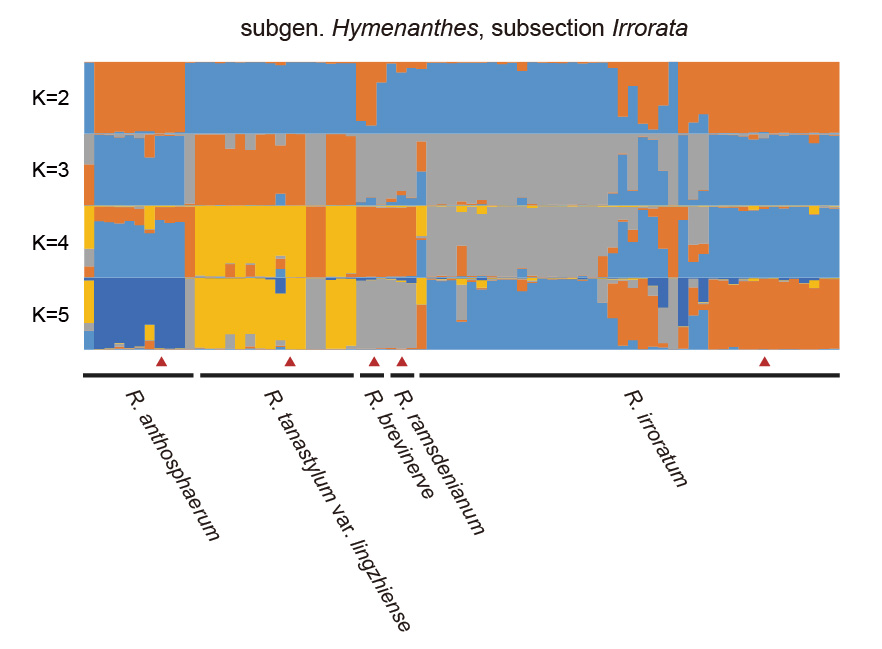
**

**Fig. S3. One example showing how to select typical samples used for whole-genome sequencing for each species.** Bar-plots revealed the genetic structure for species within subsect. *Irrorata* of subgenus *Hymenanthes*, inferred based on 15 SSR loci with different K value. Each vertical bar represents a single individual, and the height of each color represents the probability of assignment to that cluster. Individuals chosen for genome re-sequencing were marked by triangles. It should be noted that many hybrids or individuals with recent introgression occur widely in natural populations of each ‘morphologically similar’ species.

**
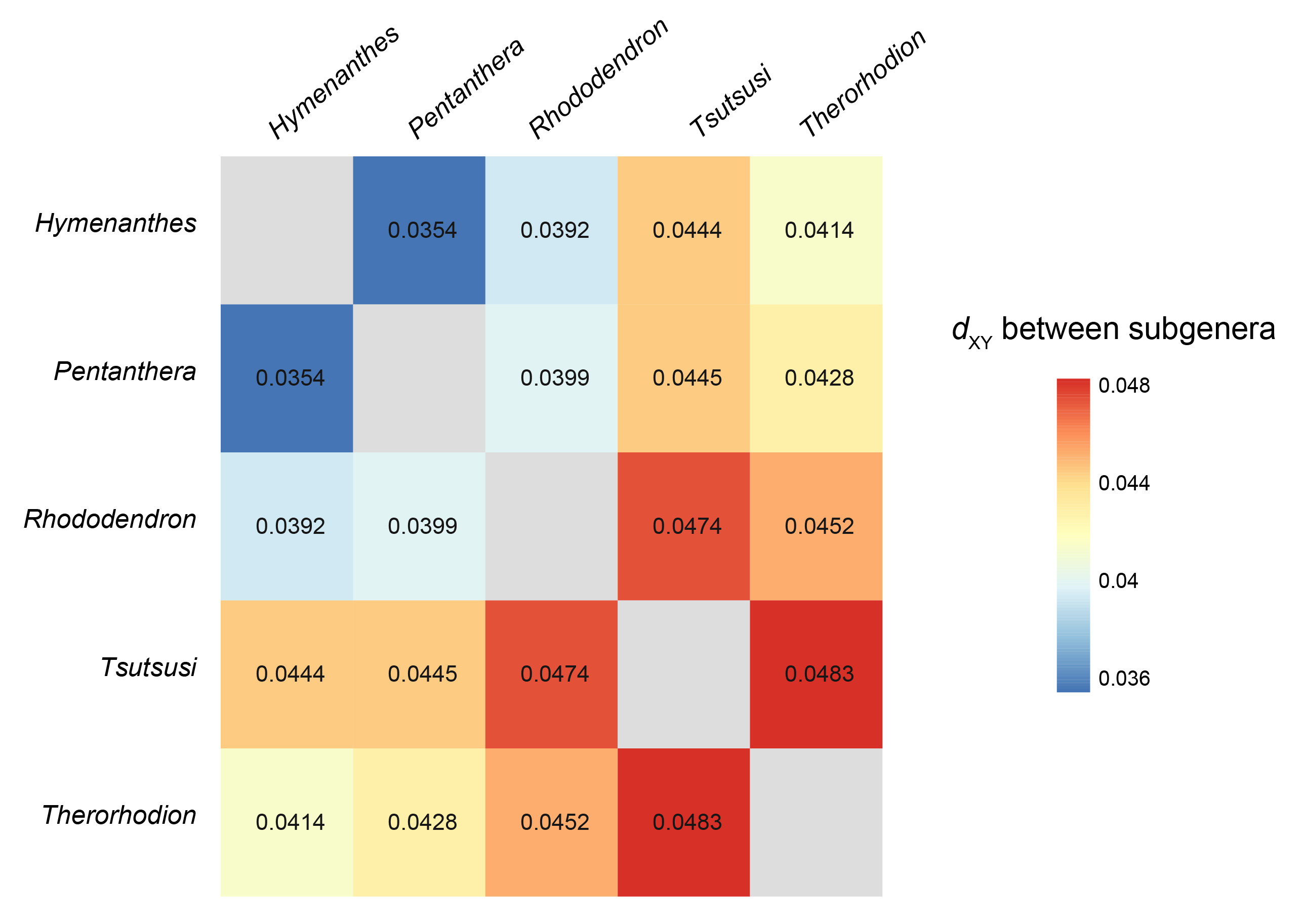
**

# Fig. S4. Average sequence divergences (*d*_XY_) between five subgenera.

**
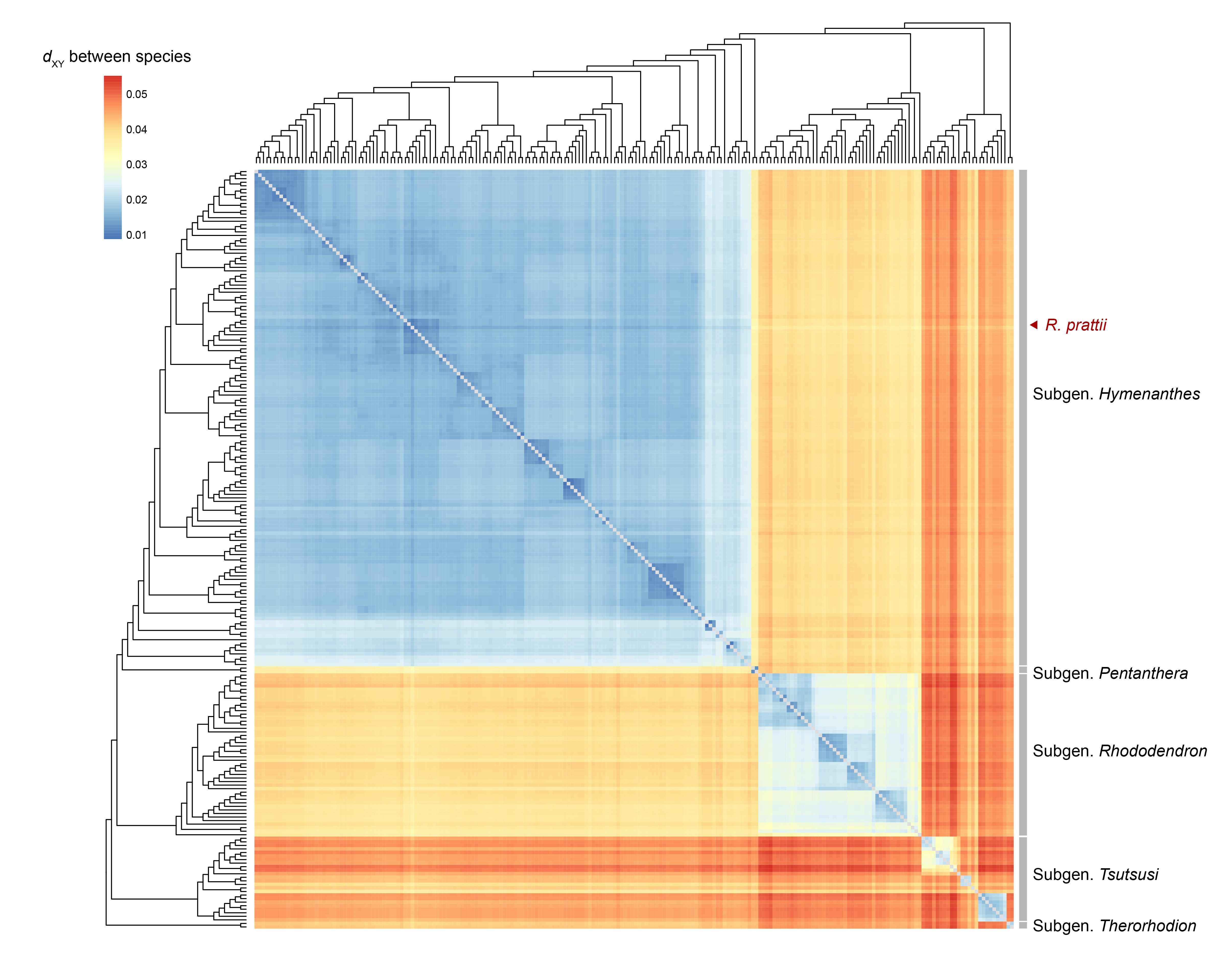
**

# Fig. S5. Average sequence divergences (*d*_XY_) between all genome sequenced species of genus *Rhododendron*. The reference species, *R. prattii*, was mark with red triangle.

**
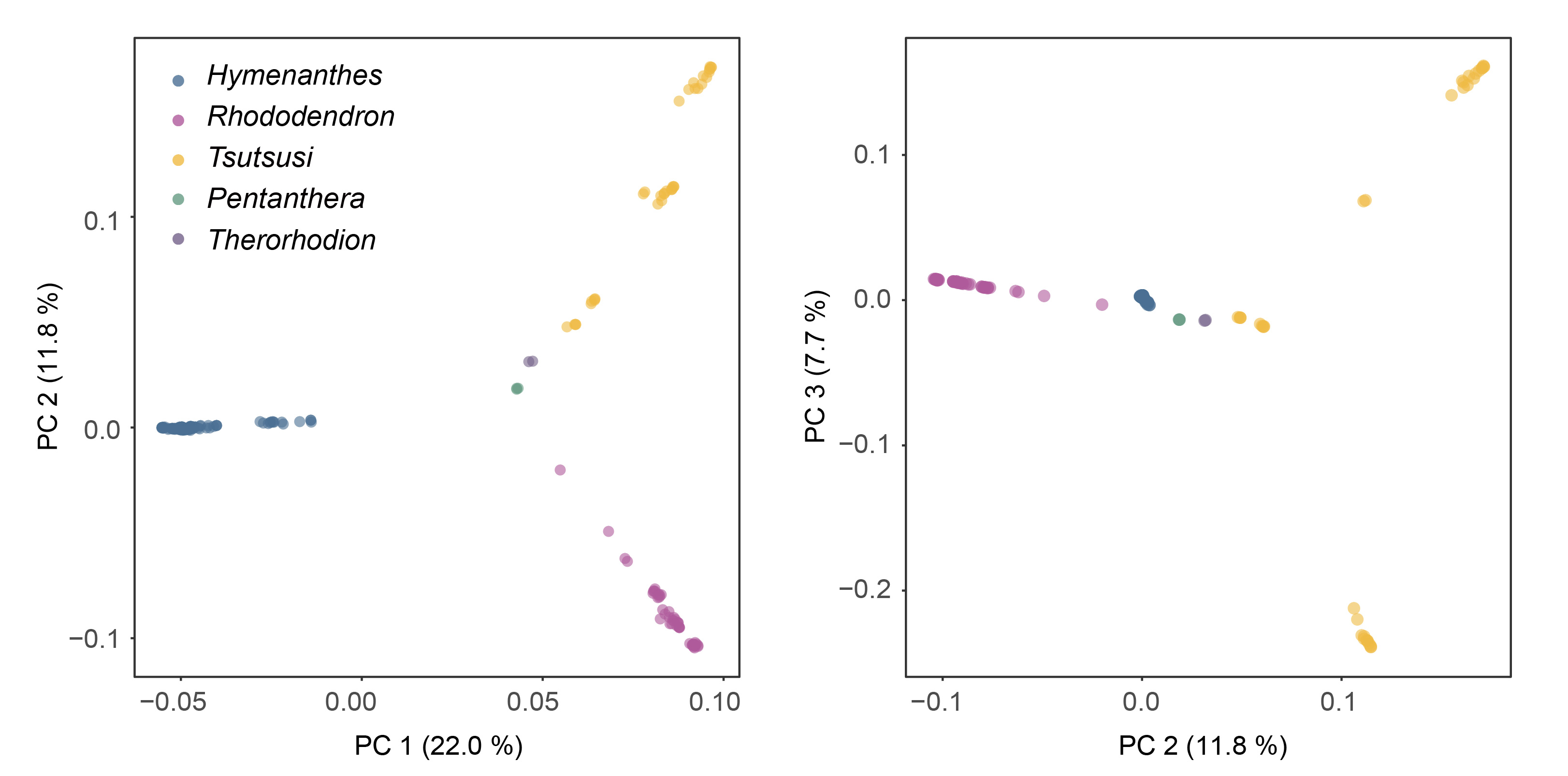
**

# Fig. S6. Principal component analysis (PCA) of whole-genome variation data, indicating the relative genomic separation of subgenus *Hymenanthes* from four other subgenera.

**
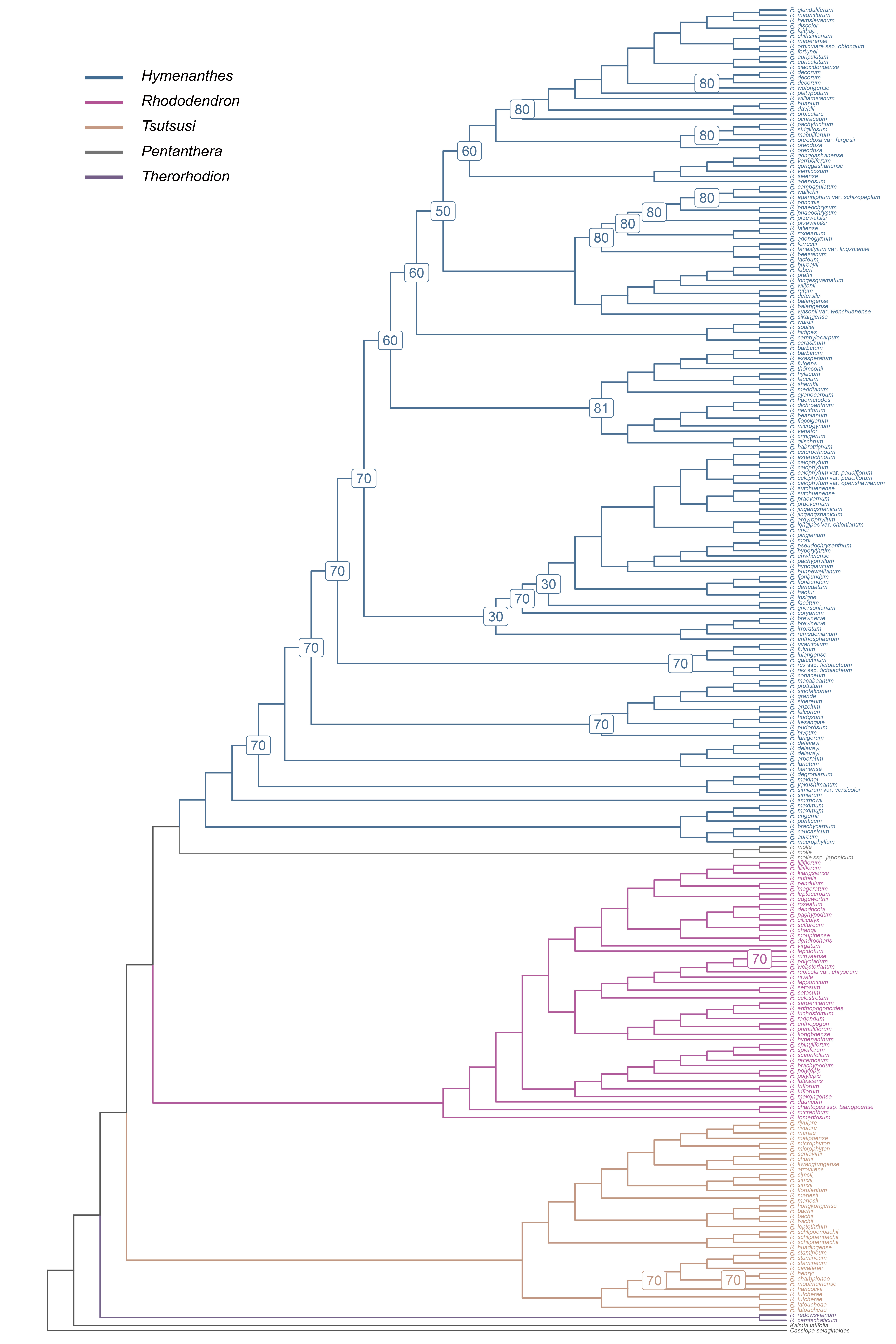
**

# Fig. S7. Tree topology estimated using the concatenation method RAxML from the whole-genome SNPs, indicating the monophyly of subgenus *Hymenanthes* sister to subgenus *Pentanthera.* Bootstrap support values >=90% were not shown in the phylogeny.

**
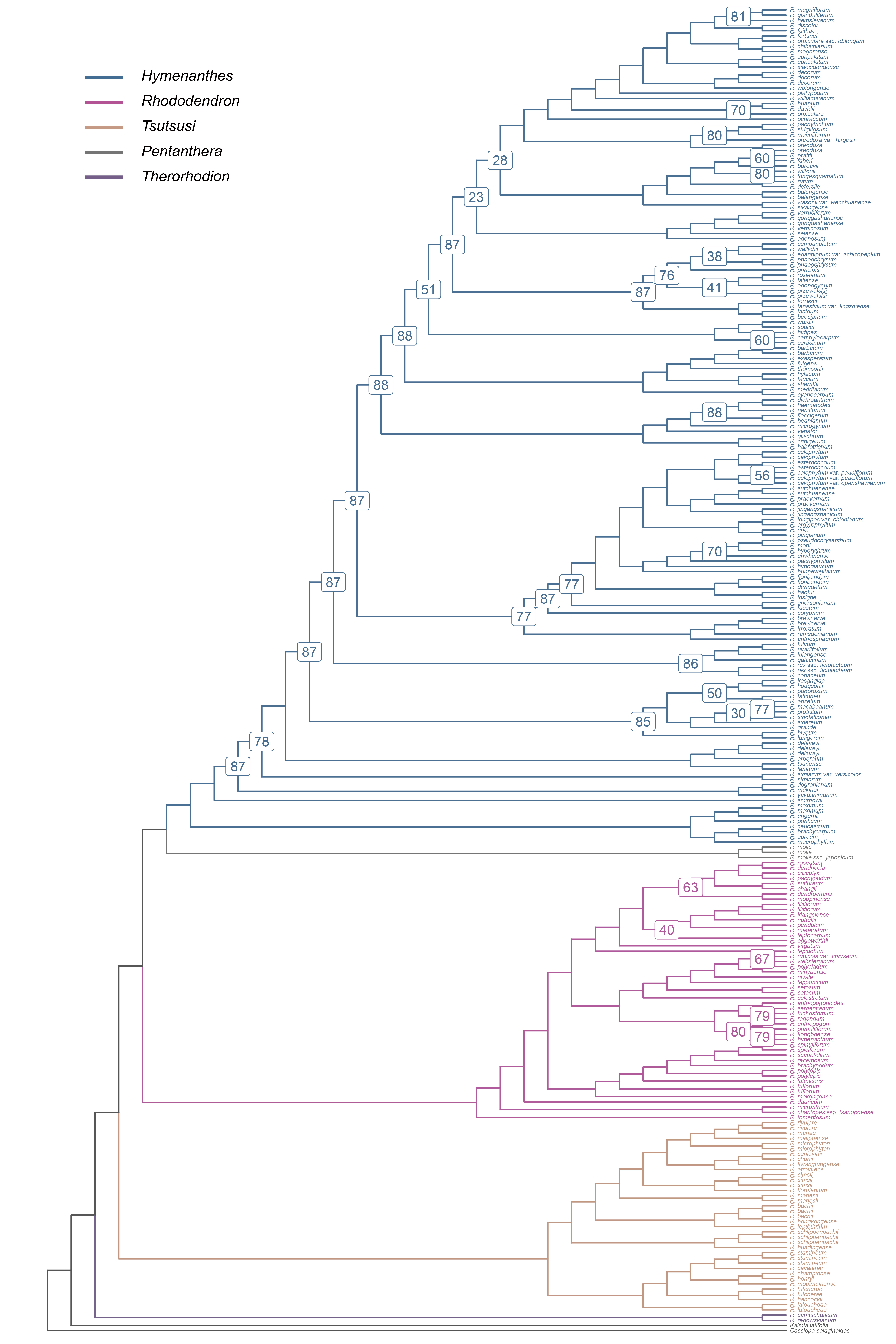
**

# Fig. S8. Tree topology estimated using the concatenation method of RAxML from the CDS dataset, indicating the monophyly of subgenus *Hymenanthes* sister to subgenus *Pentanthera*. Bootstrap support values >=90% were not shown in the phylogeny.

**
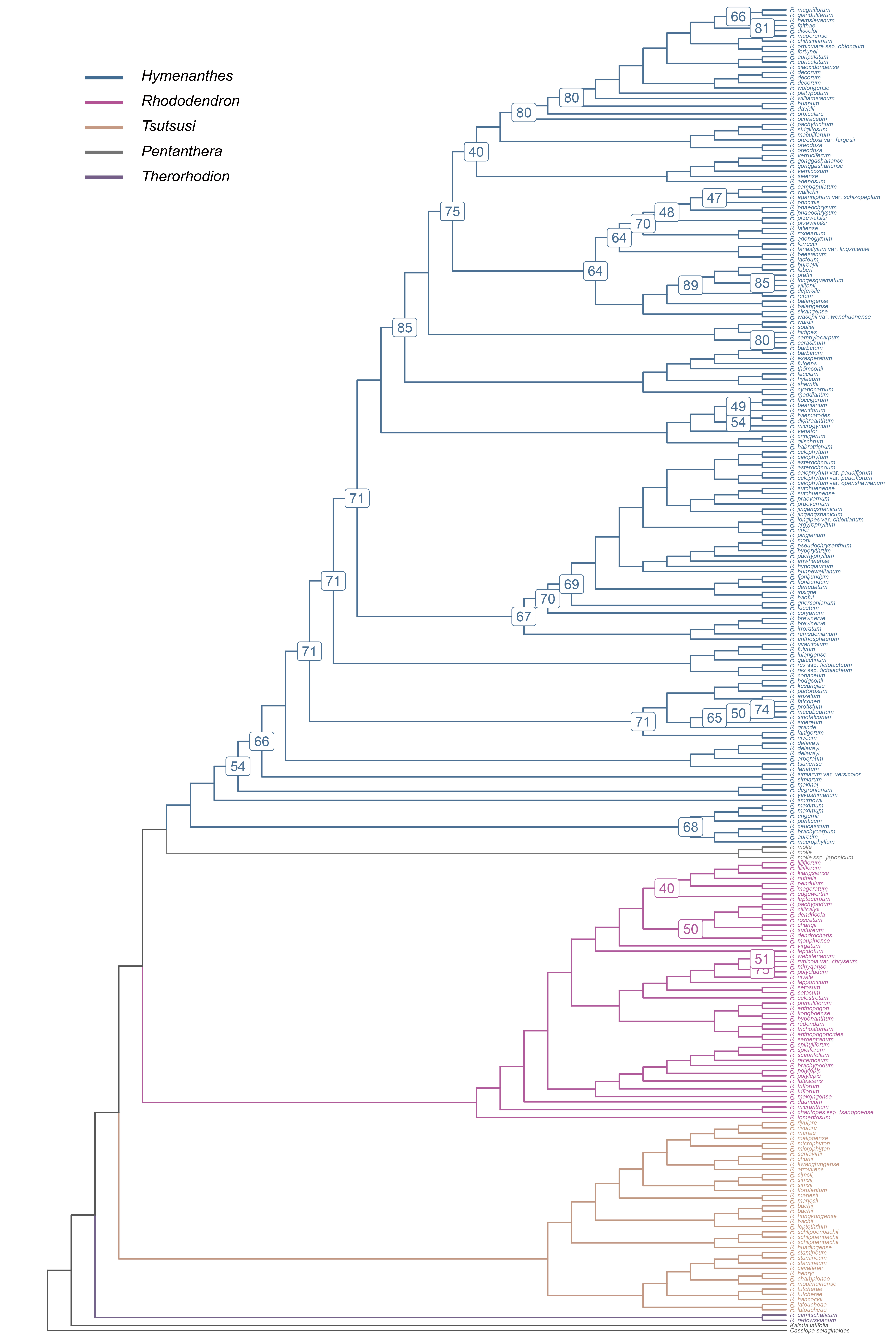
**

# Fig. S9. Tree topology estimated using the concatenation method of RAxML from the Codon12 dataset, indicating the monophyly of subgenus *Hymenanthes* sister to subgenus *Pentanthera*. Bootstrap support values >=90% were not shown in the phylogeny.

**
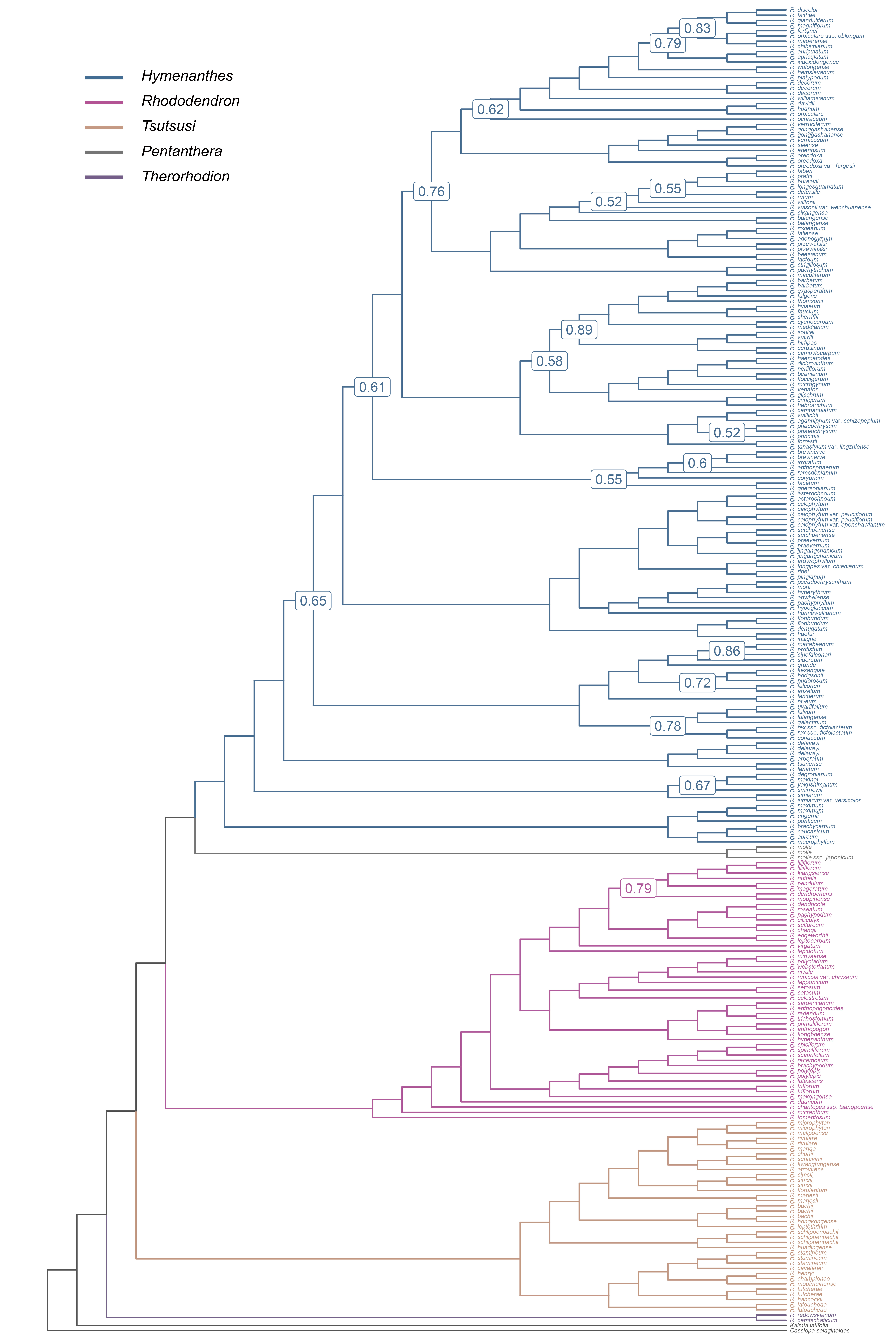
**

# Fig. S10. Tree topology estimated using the coalescent method ASTRAL from the CDS dataset, indicating the monophyly of subgenus *Hymenanthes* sister to subgenus *Pentanthera*. Numbers on nodes (support values) is local posterior probabilities. Posterior probabilities above 0.9 were not shown in the phylogeny.

**
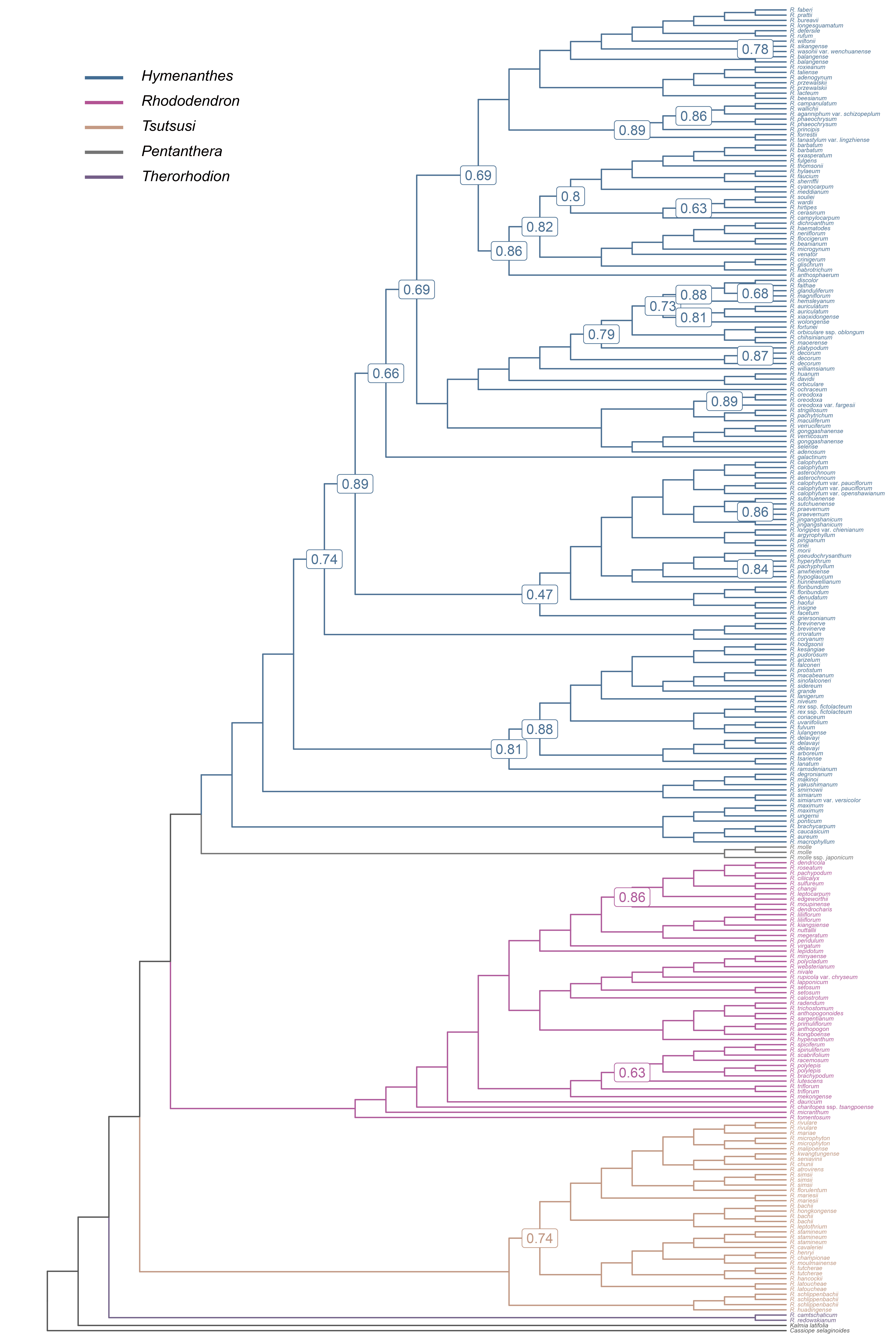
**

# Fig. S11. Tree topology estimated using the coalescent method ASTRAL from Codon12 dataset, indicating the monophyly of subgenus *Hymenanthes* sister to subgenus *Pentanthera*. Numbers on nodes (support values) is local posterior probabilities. Support values above 0.9 were not shown in the phylogeny.

**
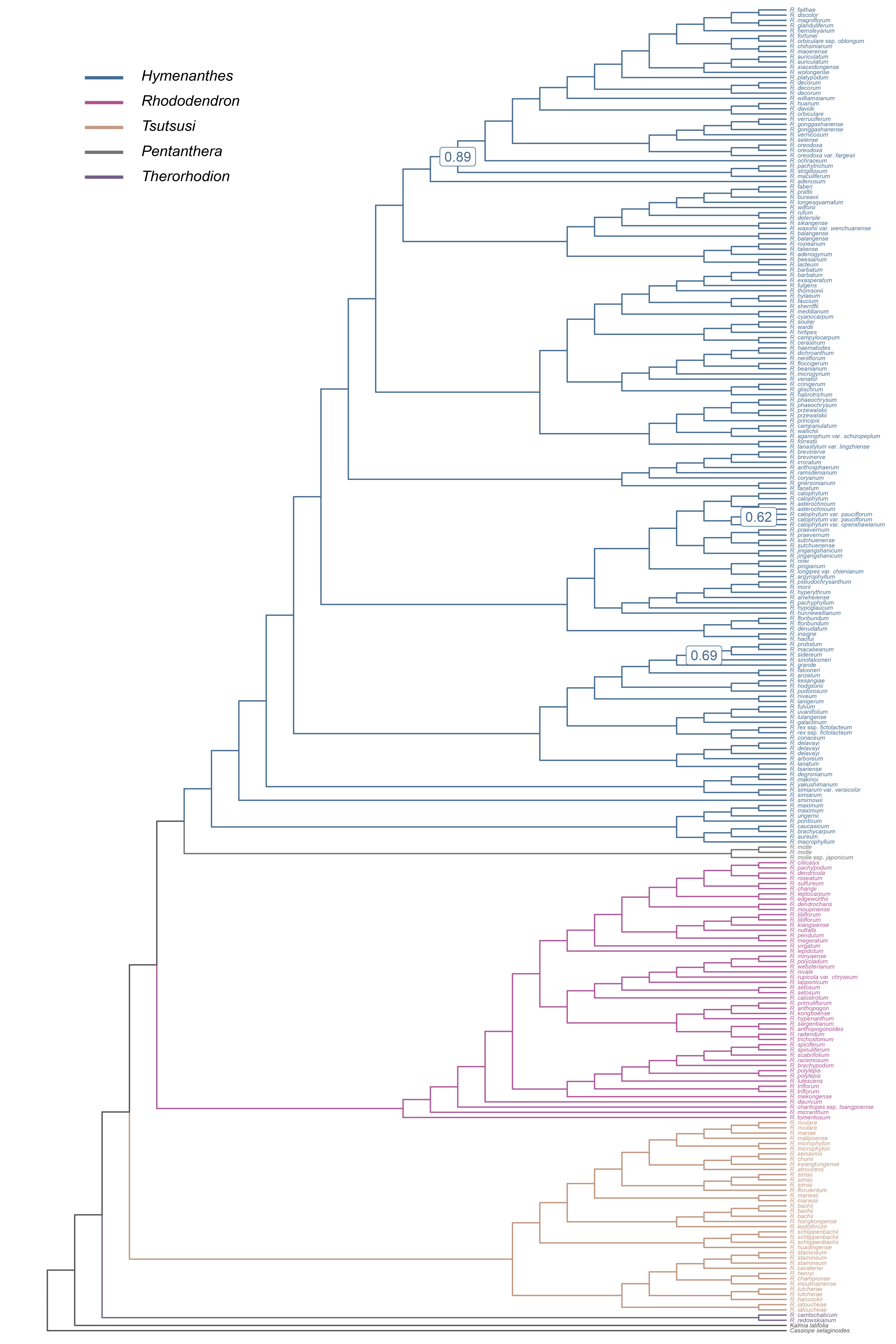
**

# Fig. S12. Tree topology inference under the ASTRAL multispecies coalescent based on 1k SNPs non-overlapping windows, indicating the monophyly of subgenus *Hymenanthes* sister to subgenus *Pentanthera.* Numbers on nodes (support values) is local posterior probabilities. Support values above 0.9 were not shown in the phylogeny.

**
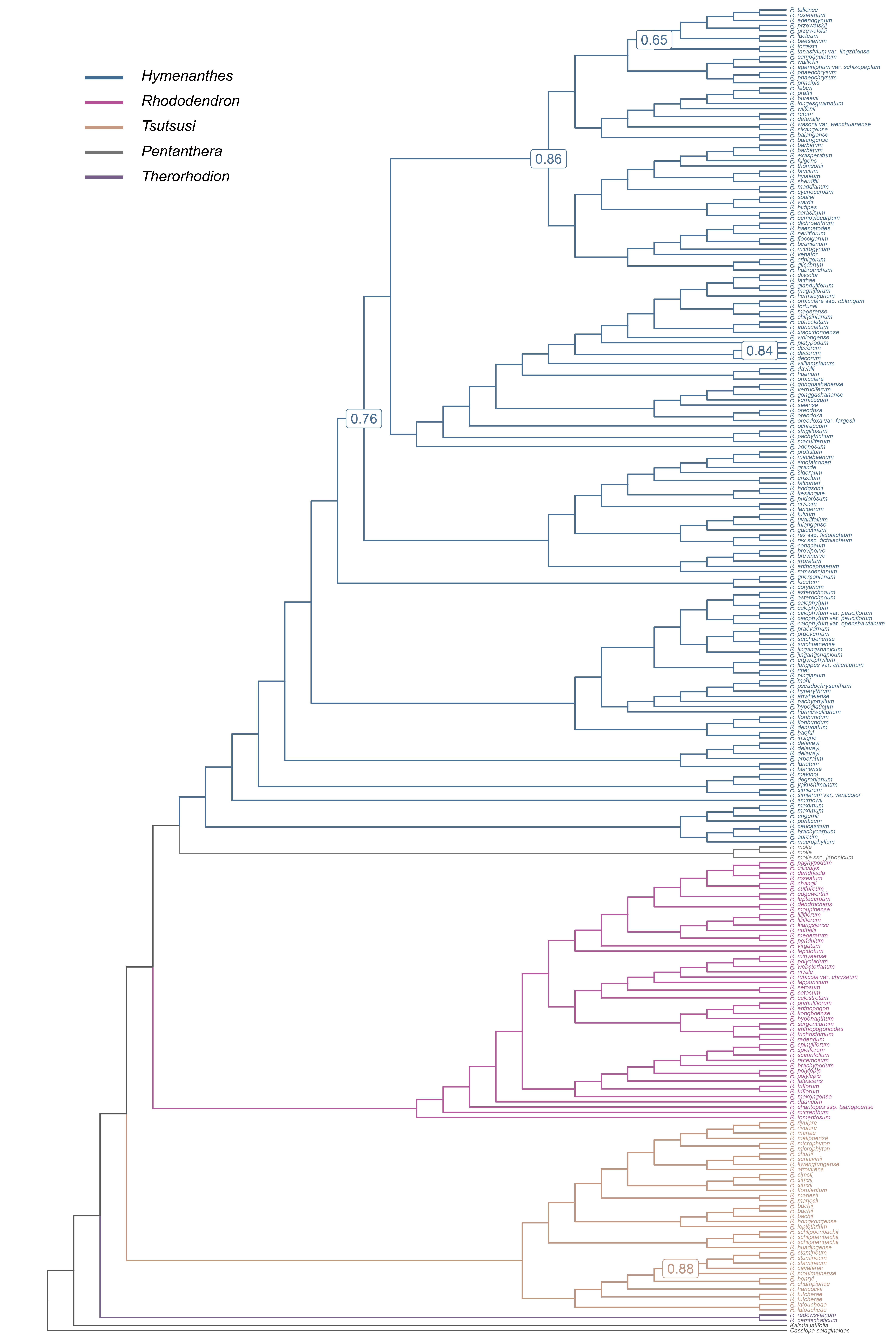
**

# Fig. S13. Tree topology inference under the ASTRAL multispecies coalescent based on 5k SNPs non-overlapping window, indicating the monophyly of subgenus *Hymenanthes* sister to subgenus *Pentanthera.* Numbers on nodes (support values) is local posterior probabilities. Support values above 0.9 were not shown in the phylogeny.

**
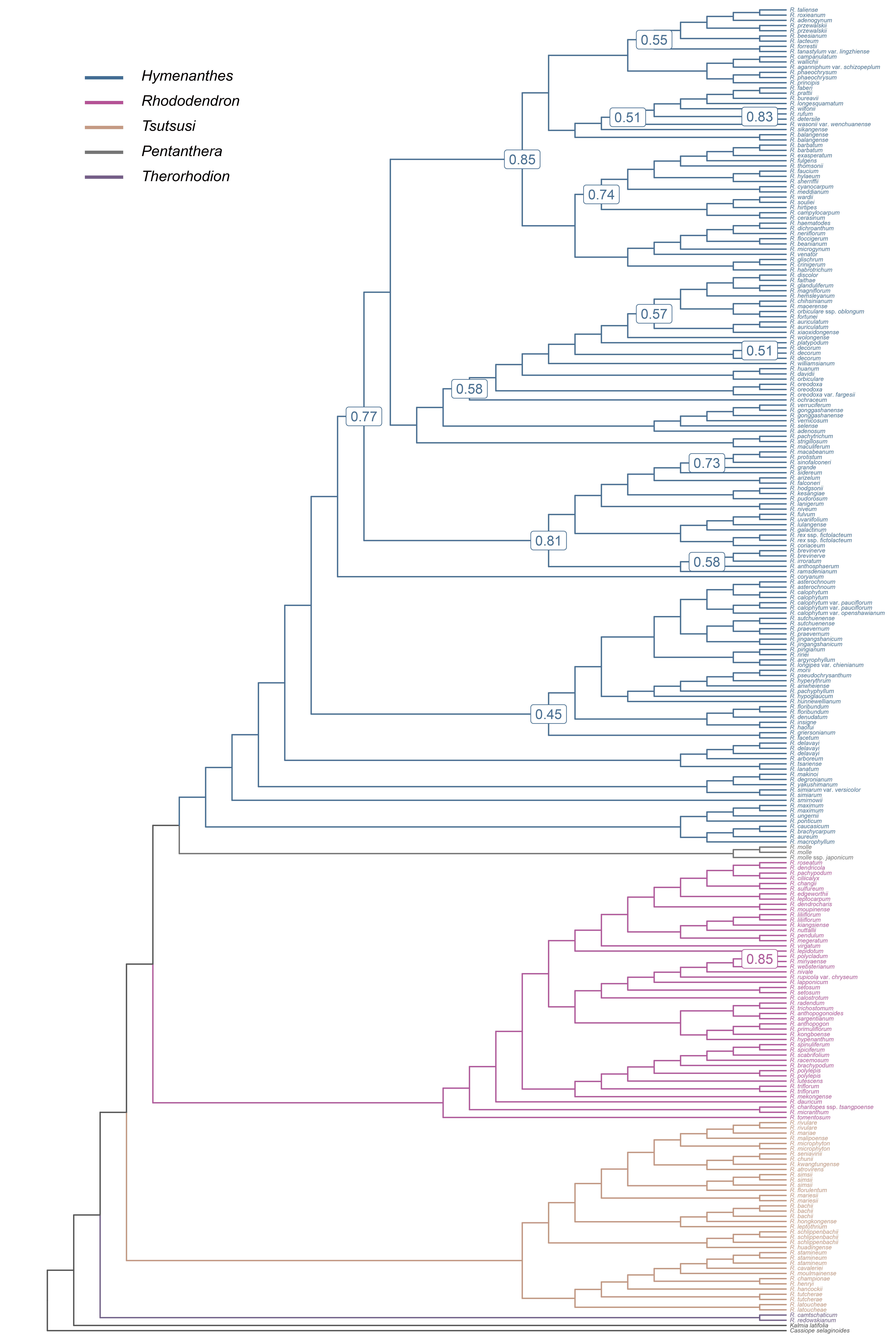
**

# Fig. S14. Tree topology inference under the ASTRAL multispecies coalescent based on 10k SNPs non-overlapping window, indicating the monophyly of subgenus *Hymenanthes* sister to subgenus *Pentanthera.* Numbers on nodes (support values) is local posterior probabilities. Support values above 0.9 were not shown in the phylogeny.

**
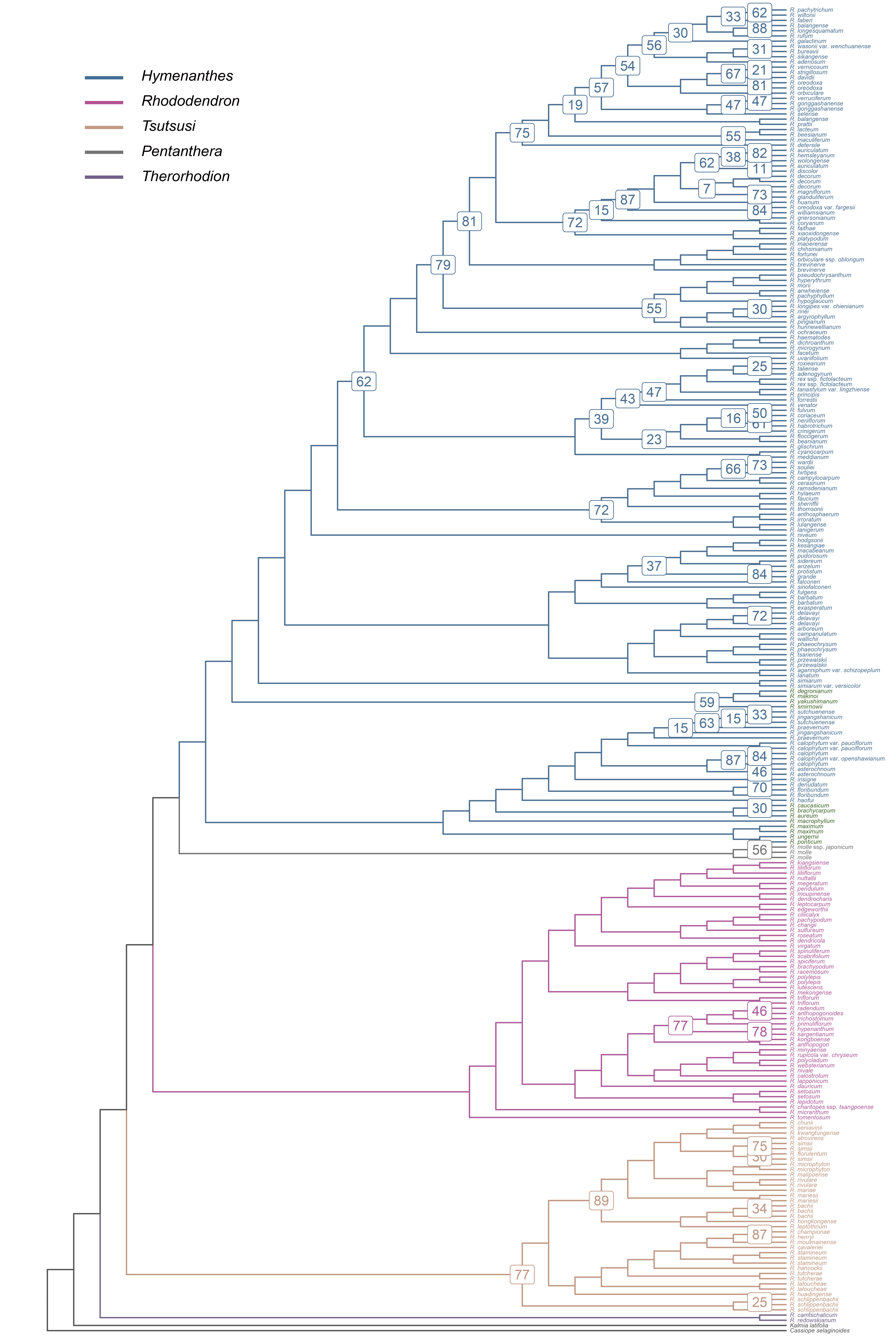
**

# Fig. S15. A maximum-likelihood chloroplast DNA phylogeny constructed with the whole genus SNPs on the chloroplast genome, indicating the monophyly of subgenus *Hymenanthes* sister to subgenus *Pentanthera.* Bootstrap support values >=90% were not shown in the phylogeny. Names of *Hymenanthes* species from Tertiary relict regions were marked in green.


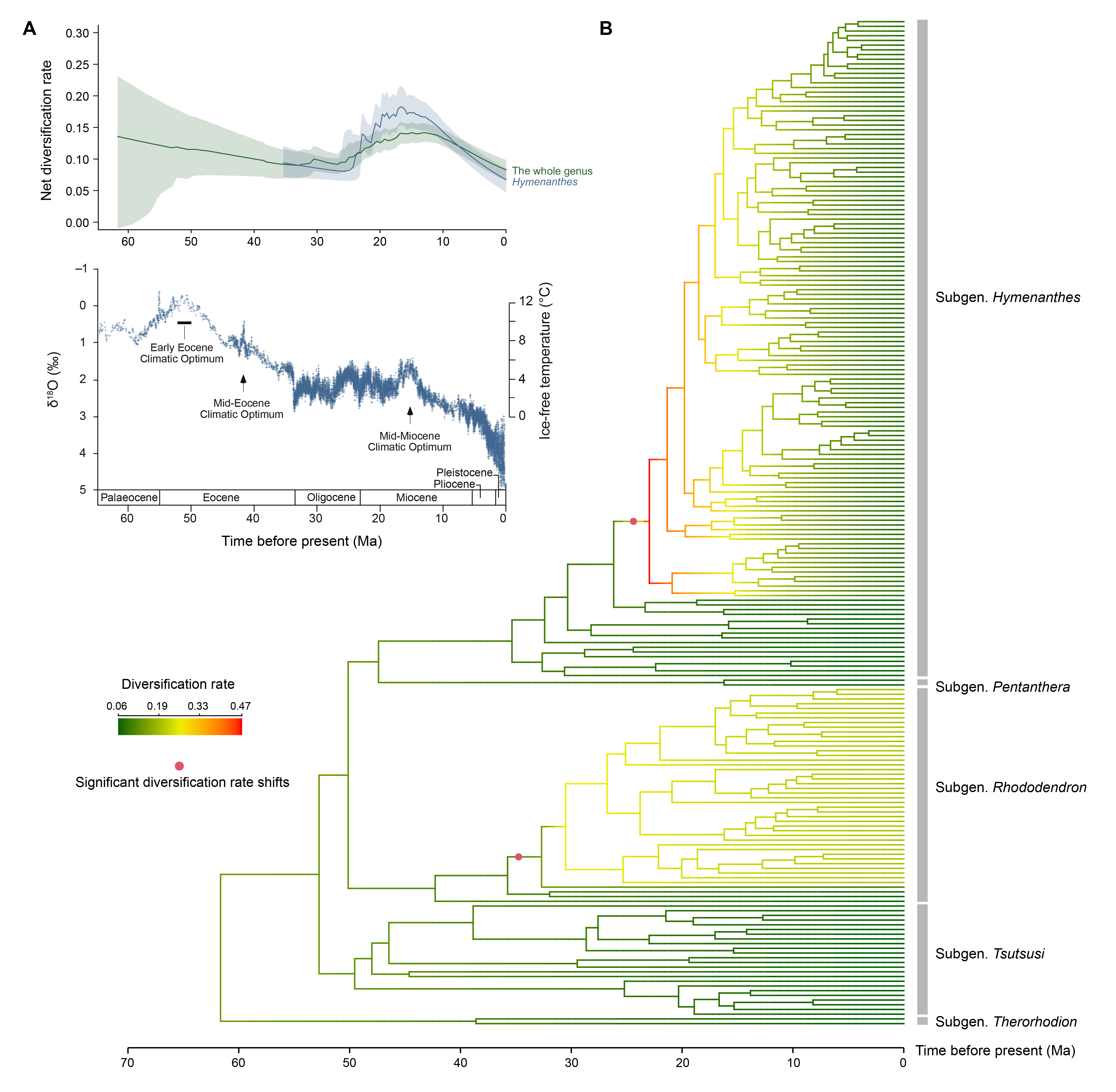


# Fig. S16. Diversification dynamics of subgenus *Hymenanthes*. (A) Net diversification rate through time for subgenus *Hymenanthes* and the whole genus and comparison with the global climate change over the last 65 million years (modified from Zachos *et al*.) [1]. Solid lines indicate median values and shaded regions indicate 90% credibility interval. (B) Diversification rate shift in the subgenus. Colors of branches show the mean diversification rate (species/million years) from BAMM.


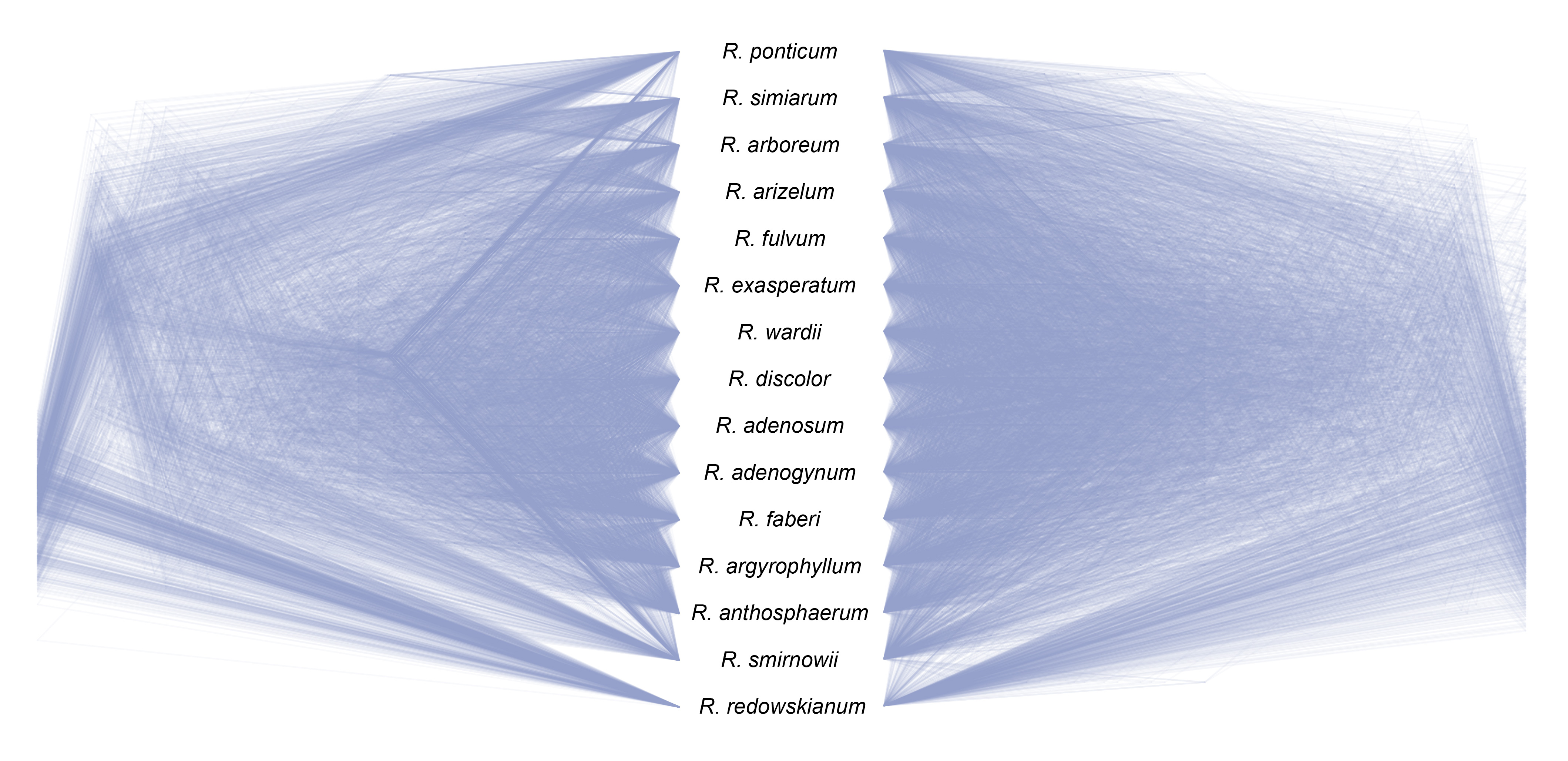


# Fig. S17. Topological conflicts among 1000 5k-SNP window trees (left) and 1000 gene trees (right). The maximum-likelihood trees were built with all sampled species and then subsampled for display to 14 individuals representing each clade or subclade of subgenus *Hymenanthes*.

**
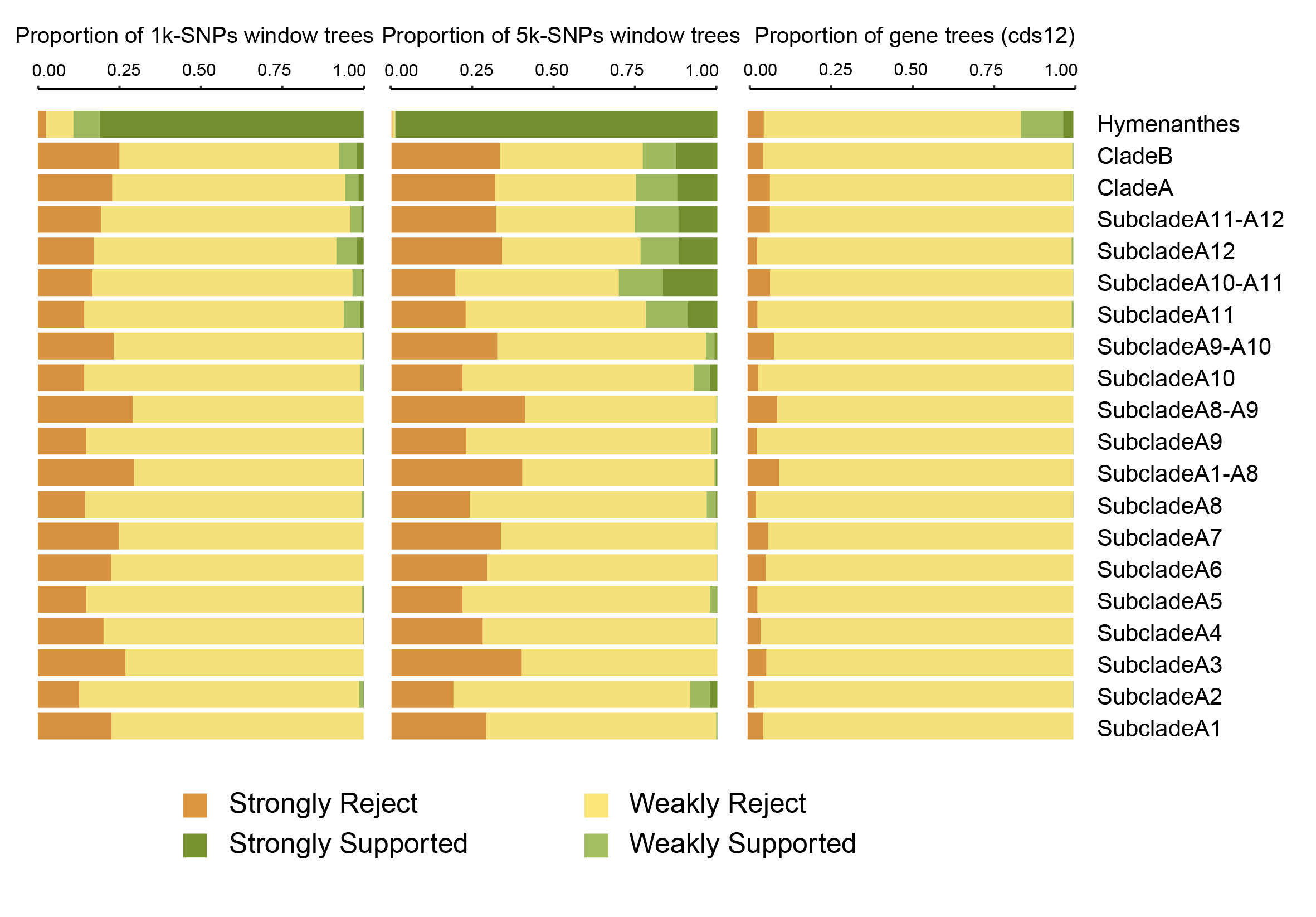
**

# Fig. S18. Local SNP window tree (1k-SNPs and 5k-SNPs) and gene tree (cds12) discordance. Local SNP window tree and gene tree compatibility revealed by the portion of trees for which clades (y-axis) are highly (weakly) supported or rejected. Weakly rejected clades are those not in the tree but being compatible if low support branches (< 75%) are contracted.





# Fig. S19. Reticulation Index for nodes in the maximum-likelihood phylogeny of subgenus *Hymenanthes* inferred from whole-genome SNPs.

**References**

1. Zachos JC, Dickens GR, Zeebe RE, An early Cenozoic perspective on greenhouse warming and carbon-cycle dynamics. *Nature* 2008;**451**:279–283.
