# Supplementary material for: Pervasive hybridization during evolutionary radiation of Rhododendron subgenus Hymenanthes in mountains of southwest China
Source: Natl Sci Rev. 2022 Dec 2;9(12):nwac276. doi: 10.1093/nsr/nwac276 (PMC9844246; doi:10.1093/nsr/nwac276)
Supplement: nwac276_Supplemental_Files [file nwac276_supplemental_files.zip › Supplementary_Data-Methods_and_Materials.docx]

Pervasive hybridization during evolutionary radiation of *Rhododendron* subgenus *Hymenanthes* in mountains of southwest China

Yazhen Ma^1,2^†, Xingxing Mao^1^†, Ji Wang^1^†, Lei Zhang^1^, Yuanzhong Jiang^1^, Yuying Geng^1^, Tao Ma^1^, Liming Cai^3^, Shuangquan Huang^4^, Pete Hollingsworth^5^, Kangshan Mao^1^, Minghui Kang^1^, Yiling Li^1^, Wenlu Yang^1^, Haolin Wu^1^, Yang Chen^1^, Charles C. Davis^3^,Nawal Shrestha^2^, Richard H. Ree^6^, Zhenxiang Xi^1^, Quanjun Hu^1^*, Richard I. Milne^5,7^*, Jianquan Liu^1,2^*

*Corresponding authors. Email: liujq@nwipb.cas.cn; r.milne@ed.ac.uk; huquanjun@scu.edu.cn.

**Methods and Materials**

**Genome sequencing**

Total DNAs for genome sequencing were extracted from fresh leaves of *R.* *prattii* of subgenus *Hymenanthes*. We sequenced the genome by integrating single-molecule real-time (SMRT) sequencing, Illumina sequencing, 10x Genomics and high-throughput chromosome conformation capture (Hi-C mapping) techniques. The SMRTbell library with an insert size of 20kb was constructed and sequenced on the PacBio Sequel system according to the manufacturer’s instructions (Pacific Bioscience). A paired-end sequencing library with an insertion size of 350bp was generated following the manufacturer’s protocol (Illumina) and sequenced using the Illumina HiSeq platform. For 10x Genomics, the barcode sequencing library was constructed on the GemCode Instrument, using the Chromium Genome Reagent Kit (10x Genomics), and sequenced with 150bp paired-end reads on the Illumina HiSeq platform. For Hi-C library construction, DNAs from tender leaves were fixed with formaldehyde, and HindIII was used to digest the cross-linked DNAs. Following ligation, purification, and fragmentation, chimeric fragments were then processed into paired-end sequencing libraries which were further sequenced using the Illumina HiSeq platform.

**Genome assembly**

The genome size and the level of genome-wide heterozygosity were estimated using findGSE v0.1 [1], based on the *k*-mer frequency distribution. De novo genome assembly was primarily carried out using PacBio SMRT long reads. Subreads correction and contigs assembly were performed using Falcon [2]. The resulting primary assembly was then polished using Quiver based on the arrow algorithm [3]. Illumina short reads were recruited for further error correction by the Pilon program [4]. Because of the high heterozygosity of *R. prattii* suggested by *k*-mer analysis, we further used Purge Haplotigs to improve the draft assembly by removing the allelic haplotypes [5]. Using BWA-mem [6], the 10x Genomics data were mapped to the contig assembly. Based on the mapping result, the primary contigs were extended into scaffolds using fragScaff (Version 140324) [7]. For further construction of chromosome-level assembly, the quality of Hi-C data was evaluated by Hic-Pro [8]. Valid interaction pairs of reads were mapped onto the scaffold assembly for correction. These corrected scaffolds were then clustered and anchored into chromosomes using LACHESIS software [9]. The completeness of the genome assembly was evaluated using BUSCO (Benchmarking Universal Single-Copy Orthologs) v 5.3.2 with the Embryophyta odb10 database [10].

**Transcriptome sequencing and data processing**

RNA samples were extracted from eight tissue types (leaf, petal, pistil, stamen, pedicel, stem, root and calyx) of *R. prattii*. For each sample, a cDNA library was constructed and sequenced using the Illumina platform. After an initial data quality control, we assembled the sequence reads into cDNA using Trinity v2.5.1 [11], in both genome-guided and *ab initio* mode. The cDNA assemblies were subsequently used to guide gene parameter construction and gene prediction. To further improve the gene prediction, we also performed full-length transcriptome sequencing for *R. prattii*. Total RNA from different tissues were mixed equally for the PacBio library construction. Three libraries with different cDNA fraction lengths (1–2 kb, 2–3 kb, and >3 kb) were constructed according to the manufacturer's instruction and subsequently sequenced on the PacBio Sequel sequencer platform. The raw Iso-Seq reads were processed using SMRTlink software to obtain polished consensus sequences. Based on the Illumina RNA-seq data, additional nucleotide errors were corrected by LoRDEC [12]. Finally, the high-quality transcriptome sequences were then mapped to the reference genome assembly using the Genomic Mapping and Alignment Program (GMAP) [13]. The Python script collapse_isoforms_by_sam.py from the PacBio repository (https://github.com/PacificBiosciences/cDNA_primer) was used to predict transcript structures from the GMAP result and to remove redundant transcripts for subsequent gene prediction**.**

**Repetitive element and gene annotation**

Whole-genome transposable element (TE) annotation was performed using the Extensive *de-novo* TE Annotator (EDTA) pipeline [14]. Prediction of gene structure was conducted with a combination of multiple prediction methods, mainly homologous prediction, transcriptome-based prediction and de novo prediction. For homology-based prediction, protein sequences from nine related species, *Actinidia chinensis*, *Vaccinium macrocarpon*, *Coffea canephora*, *Camellia sinensis*, *Daucus carota*, *Primula veris*, *Solanum lycopersicum*, *Arabidopsis thaliana* and *Vitis vinifera* were mapped onto the *R. prattii* genome using Exonerate v2.4.0 [15] to search the homology alignments. In the transcriptome-based prediction, assembled transcripts were aligned to the genome assemblies using PASA (Program to Assemble Spliced Alignment) v2.1.0 (http://pasapipeline.github.io/), and gene structures information obtained from IsoSeq reads were also integrated into the transcript evidence. On the basis of mapping location, valid transcript alignments were clustered and assembled into gene structures. The high-quality gene models obtained from transcriptome-based prediction were also selected as the training set for AUGUSTUS v3.2.3 [16] and GlimmerHMM v3.0.4 [17], which were used for de novo prediction depending on the statistical characteristics of genome sequence data. All candidate genes models generated by the three approaches were integrated into a consensus set using EvidenceModeler v1.1.1 (https://evidencemodeler.github.io/). Finally, we used PASA to update the EVM annotation based on transcriptome assembly, and added the UTRs information to the final gene set. Putative gene functions were annotated based on comparisons with the NCBI Non-Redundant database, TrEMBL and SwissProt databases using blastp v2.7.1. Protein domains of genes were determined using InterProScan v5.31.70 [18] against InterPro protein databases. Gene Ontology terms and KEGG pathway information for each gene were assigned by Blast2GO v2.5 [19] and KAAS (https://www.genome.jp/kegg/kaas/), respectively. Orthologous groups of *R. prattii* and other five *Rhododendron* species (*R. delavayi, R. griersonianum, R. henanense, R. simsii and R. ovatum*) with high-quality genome assemblies and annotations were constructed on the basis of the best bidirectional hits using OrthoMCL v2.0.95 (https://orthomcl.org).

**Materials collection and species selection for genome-scale radiation analysis**

*Rhododendron* is recognized as one of the most taxonomically challenging plant genera due to rampant hybridization [20–22] during rapid radiation. We examined and collected materials from around 20,000 individuals comprising ca. 800 populations of 292 species of subgenus *Hymenanthes*, plus ca. 400 natural populations of 140 species from four other *Rhododendron* subgenera (taxonomic positions of some species are disputed between different taxonomists). Nearly all of these collections derived from the mountains of southwest China and surrounding regions, the exceptions being fifteen species from eastern Asia, four from southwestern Eurasia and three from North America. One of authors as a taxonomic expert on *Rhododendron* (Prof. Yuying Geng) examined our specimens and determined which species were morphologically distinct (i.e. had clear morphological gaps from other species). We tried our best to use morphological methods to identify ‘good species’ with stable morphological gap and stability between multiple individuals at population level (multiple sites) for further analysis. Next, to detect and exclude material affected by recent introgression, we genotyped 1620 samples from 171 widespread *Rhododendron* species (103 of them from subgenus *Hymenanthes*) using 15 pairs of nuclear SSR markers, chosen for their ability to distinguish between closely related species, and identify cryptic introgression [20]. Total genomic DNA of dry leaf material was extracted using a modified CTAB method [23]. After a 5-min denaturation at 95°C, PCR was performed for 35–36 cycles (95°C for 45 s, 55°C for 40 s, and 72°C for 80 s), with a 7-10 min final extension at 72°C. The resulting PCR products were differentiated and genotyped on an ABI 3830xl DNA analyzer (Applied Biosystems, Foster City, CA, USA). To detect signs of recent introgression at the genome level for each species, we carried out clustering analyses for genotyped individuals using STRUCTURE version 2.3.4 (https://web.stanford.edu/group/pritchardlab/structure.html). Samples of species within the same subsection or section were selected to constitute an analysis unit. For each dataset, twenty independent runs were carried out with different *K* (the number of clusters) values. All runs involved 1,600,000 Markov chain Monte Carlo repetitions with burn-in set to 800, 000. For each *K*, the result with the minimum value of LnP(D) was chosen as the best one. Individuals that lacked signs of recent introgression were selected for whole-genome sequencing. Despite these time-consuming efforts, we still could not assure that all used materials could represent ‘true species’ as independently evolving lineages. For example, because of multiple origins and probable clonal reproduction, F1 hybrids constitute a ‘distinct species’, *R. agastum*, with ‘morphological distinction and stability’ from two parental species at the ‘population’ level based on previous study [24]. We further adopted this species as one control for initial analyses to identify more such likely ‘species’ using whole-genome sequencing data (see below).

**Sequencing and variant calling for radiation analysis**

DNA samples from 277 individuals of 236 morphologically well-delimited species (164 individuals of 143 species for subgenus *Hymenanthes*) covering all subsections of *Hymenanthes*, its sister group subgenus *Pentanthera*, and three other major evolutionary lineages of genus *Rhododendron* distinguished by previous studies [25], were used for the construction of Illumina sequencing libraries. For each sample, we sequenced 150-bp paired-end reads on an Illumina HiSeq platform. After an initial quality control, clean reads were aligned to the *R. prattii* reference genome using the BWA-mem algorithm with default options. Duplicate reads were marked and removed using the MarkDuplicates tool from the Picard software package (https://github.com/broadinstitute/picard) with default options. Based on the relative frequency of each allele sequenced at heterozygous positions throughout the genome, the ploidy of each sample was determined using the R package vcfR (https://github.com/knausb/vcfR). From this, we further excluded 21 individuals of 19 putative polyploid species within subgenus *Rhododendorn*, from the final analyses. A total of 256 bam files, each from one individual, of 217 diploid species (including 143 from subgenus *Hymenanthes*) were used to call the variants for the whole genus using ‘HaplotypeCaller’ and ‘GenotypeGVCFs’ in GATK v.3.8. For the whole genus, two different versions of the VCF file were created: one where all sites were called (backgroup) and one with only variable sites. For the all-site dataset, genotypes with reads coverage <5 were assigned as missing and sites with > 20% missing genotype for all samples were removed. For the variant filtering, we first excluded all indels, and also sites within 5 bp from any indels, and restricted all remaining variant sites to biallelic. Then hard filtering was performed on the dataset by removing sites determined to be of low quality (QUAL<50, QD < 2.0, FS > 40.0, MQ < 40.0, MQRankSum < −12.5, ReadPosRankSum < −8.0). We also assigned genotypes as missing if their reads coverages were extremely low (<8) or high (>120). Sites with missing genotype count percentage exceeding 5% of the sample size were excluded from the final SNPs datasets. In addition, whole genomes of two species (*Cassiope selaginoides* and *Kalmia latifolia*) from related genera of Ericaceae were also sequenced with high depth, and added to the SNPs dataset as outgroups in the phylogenetic analysis. For gene flow analysis, an independent SNP dataset was generated from the samples of subgenus *Hymenanthes* with the same procedure of the whole genus SNPs. Genotype refinement, imputation, and phasing of the variant sites were implemented in BEAGLE v4.0 [26].

The average sequence divergence (*d_XY_*) between species was estimated using Pixy v1.2.7 [27] in 100 kb non-overlapping windows. We also calculated the genomic sequence diversity within individuals (heterozygosity) of each *Hymenanthes* species to exclude those likely ‘false species’ consisting of F1 hybrids like *R. agastum* [24]. We found that the heterozygosities of *R. agastum* and other twos, *R. catawbiense* and *R. adenopodum*, were obviously higher than those of the other sampled species as special outliers. Therefore, the sampled individuals of *R. catawbiense* and *R. adenopodum* may also comprise F1s as *R. agastum* [24]. We then excluded these three species in the following analyses. However, it should be noted that these extensive efforts that have taken a long time still could not totally exclude those likely ‘false species’ comprising F1s with ‘morphological distinction and stability’ at the ‘population’ level, if such F1 hybrids arose from two closely related species. Despite this, a few of such ‘samples’ could not affect our general conclusion based on a large-scale data analysis, because all analyses produced similar results with or without the above-mentioned likely ‘false species’ *R. agastum*, *R. catawbiense* and *R. adenopodum*. These species and their closely related species need further investigation based on population genomic data to determine their hybrid origins and compositions.

**Principal component analysis and phylogenetic analyses**

For principal component analysis (PCA) of the 214 diploid species, we discarded SNPs that had a correlation coefficient higher than 0.2 with any other SNP within a 50-SNP stepping window using PLINK v1.07 (http://zzz.bwh.harvard.edu/plink/) with parameter --indep-pairwise 50 5 0.2, and only retained SNPs with minor allele frequency ≥0.05 using the vcftools v0.1.14 (http://vcftools.sourceforge.net/). PCA on the resulting set of variants was performed using the smartpca function in Eigensoft v5.0.2 (https://github.com/DReichLab/EIG) with default parameters. Significance levels of principal components were determined using the Tracy-Widom test.

To infer the phylogenetic relationship among these 214 *Rhododendron* species, a maximum-likelihood (ML) tree of the concatenated whole-genome SNPs was constructed in RAxML using the GTRGAMMA mode. One hundred bootstrap replicates were obtained using RAxML’s rapid bootstrapping algorithm. SNPs within the single-copy orthologous genes among the six *Rhododendron* species identified above were then extracted across all sampled species and divided into two datasets, once comprising the first plus second codon positions and the other the complete coding sequences. In each dataset, only genes with high site coverage (>50%) in the all-site vcf dataset and containing at least 10 phylogenetically informative sites were used for phylogeny inference. For each gene dataset, the individual gene maximum likelihood trees were estimated using RAxML [28]. Newick utilities (https://github.com/tjunier/newick_utils) was used to collapse branches with <10% bootstrap support, within each gene tree. Then, using the resulting ML trees from each gene as input, we used ASTRAL v.5.6.1 [29], a coalescent based method, to generate the species tree. In addition, two concatenated species trees were also inferred based on the concatenated sequences of two gene datasets by RAxML. To obtain local window trees built using regions with similar strengths of phylogenetic signal, we further divided the whole-genome SNPs into multiple non-overlapping windows, each comprising the same number of SNPs. Three datasets with different window sizes (1,000, 5,000 and 10,000 SNPs) were constructed. Small windows at the end of each chromosome were discarded. Maximum likelihood phylogenies of each window dataset were also estimated using RAxML. Local window trees with weakly supported collapsed were then utilized by ASTRAL to infer species trees. To visualize the discordance of phylogenies across the genome, gene trees or local window trees were superimposed using DensiTree (http://compevol.auckland.ac.nz/software/DensiTree/) with branch length information being discarded. Local phylogeny compatibility analysis was performed using DiscoVista v.1.0 [30]. To construct a chloroplast DNA phylogenetic tree, we assembled the chloroplast genome of *R. prattii* based on Paobio-long reads and Illumina-short reads using Unicycler [31] and Canu [32]. From this, the plastome variants (*cp*SNPs) of the whole genus were then obtained under the same procedure as the nuclear genome SNPs. A maximum likelihood tree was inferred using RAxML under the GTRGAMMA model and with 100 bootstraps.

**Estimation of divergence time and diversification rate**

A Bayesian relaxed molecular clock approach was used to estimate divergence times using the program MCMCtree in the PAML package v4.9e [33]. We used the coding sequence data and the ML tree based on whole-genome SNPs as inputs for this analysis. Sequences from *Actinidia eriantha* were also added into the dataset as outgroup. The fossil of *Rhododendron newburyanum* [34] dated to the Palaeocene (c. 56 Ma) was used to set the minimum age of the *Rhododendron* crown group, whereas the maximum divergence time between Ericaceae and Actinidiaceae (125 Ma) [35] was used to calibrate the external tree. For the MCMC process, we applied a burn-in of 2,000,000 and sampled 20,000,000 MCMC generations, sampling every 200 iterations. To check convergence of the stationary distribution, two independent MCMC runs were performed for results comparison. The resulting effective sample size (ESS) for each parameter was determined using the program Tracer 1.7.1 (https://beast.community/tracer). The ESS values for all parameters were >200.

To better understand the dynamics of lineage diversification, we used BAMM version 2.5 (http://bamm-project.org/) and the R package BAMMtools to generate Bayesian inferences of diversification rate based on the time-calibrated phylogeny. Previously published estimates for the number of species within each subgenus were used to set clade-specific sampling probabilities. Priors for speciation and extinction were set empirically using the setBAMMpriors function. The geometric prior on the expected number of regime shifts was set to 1, as recommended in the BAMM documentation. We ran each BAMM Markov chain Monte Carlo analysis for 50 million generations with a sampling frequency of 1/5,000 and assessed convergence by visually inspecting plots of the likelihood trace and calculating the effective sample size after discarding the first 10% of the run as burn-in.

**Identification of shared polymorphisms and tests of historical hybridization**

To detect shared haplotypes between species, a refined identity-by-descent (IBD) blocks analysis was conducted based on whole-genome SNPs using BEAGLE with the following parameters: window = 50,000; overlap = 5,000; ibdtrim = 40; ibdlod = 3. To further detect potential interspecies gene flow under a hypothesis of ILS, we computed all possible *D*-statistics [36] for triplets among the sampled *Hymenanthes* species. For each triplet, the *D*-statistics were calculated for all three possible species topologies. *R. redowskianum* was chosen as the outgroup and only sites that had homozygous allele states in both *R. redowskianum* and *R. camtschaticum* were considered in this analysis. We used *D*_min_, the absolute value of the lowest *D*-statistic score for each triplet, to assess the overall extent of violation of the assumption of tree-like species relationships. If *D*_min_ is significantly elevated, then allele sharing within the trio of species is inconsistent with any simple tree topology [37]. Statistical significance was assessed using block jackknife on windows of 60-kb SNPs, and multiple testing was accounted for by calculating family-wise error rate (FWER) following the Holm–Bonferroni method. Based on the triplets with significantly elevated *D* scores, we further calculated the Reticulation Index for each node in the nuclear genome ML tree, according to the method from Cai *et al*. [38]. In addition, as a single gene-flow event between ancestral lineages can affect multiple contemporary species, leading to multiple introgression signals in *D*-statistics, we further used the *f_b_*(*C*) statistic [37], a summary of *f*_4_ admixture ratios, to assess the excess allele sharing between a species *C* and a branch b compared to the sister branch of b. The ML tree constructed with whole-genome SNPs was chosen as the phylogenetic framework. The *f*_4_ admixture ratio statistics were first calculated for all triplets according to the topology reflected in the species tree. Then the *f_b_*(*C*) statistic was computed as: *f_b_*(*C*) =median*_A_*[min*_B_* [*f*(*A*,*B*;*C*,*O*)]], where *B* runs over all clades that are descendants of 𝑏, and *A* are samples descending from the sister branch of *b*. To get a branch-specific measure of significance, each *f_b_*(*C*) score was also assigned an associated *z*-score as *Z_b_*(*C*)=median*_A_*[min*_B_*[Z(*A*,*B*;*C*,*O*)]].

**References**

1. Sun H, Ding J, Piednoël M *et al.* *findGSE*: estimating genome size variation within human and *Arabidopsis* using *k*-mer frequencies. *Bioinformatics* 2018;**34**:550–7.

2. Chin CS, Peluso P, Sedlazeck FJ *et al.* Phased diploid genome assembly with single-molecule real-time sequencing. *Nat Methods* 2016;**13**:1050–4.

3. Chin CS, Alexander DH, Marks P *et al.* Nonhybrid, finished microbial genome assemblies from long-read SMRT sequencing data. *Nat Methods* 2013;**10**:563–9.

4. Walker BJ, Abeel T, Shea T *et al.* Pilon: an integrated tool for comprehensive microbial variant detection and genome assembly improvement. *PLoS One* 2014;**9**:e112963.

5. Roach MJ, Schmidt SA, Borneman AR. Purge Haplotigs: allelic contig reassignment for third-gen diploid genome assemblies. *BMC Bioinformatics* 2018;**19**:460.

6. Li H, Durbin R. Fast and accurate short read alignment with Burrows-Wheeler transform. *Bioinformatics* 2009;**25**:1754–60.

7. Adey A, Kitzman JO, Burton JN *et al.* In vitro, long-range sequence information for de novo genome assembly via transposase contiguity. *Genome Res* 2014;**24**:2041–9.

8. Servant N, Varoquaux N, Lajoie BR *et al.* HiC-Pro: an optimized and flexible pipeline for Hi-C data processing. *Genome Biol* 2015;**16**:259.

9. Burton JN, Adey A, Patwardhan RP *et al.* Chromosome-scale scaffolding of de novo genome assemblies based on chromatin interactions. *Nat Biotechnol* 2013;**31**:1119–25.

10. Waterhouse RM, Seppey M, Simão FA *et al.* BUSCO applications from quality assessments to gene prediction and phylogenomics. *Mol Biol Evol* 2018;**35**:543–8.

11. Haas BJ, Papanicolaou A, Yassour M *et al.* *De novo* transcript sequence reconstruction from RNA-seq using the Trinity platform for reference generation and analysis. *Nat Protoc* 2013;**8**:1494–512.

12. Salmela L, Rivals E. LoRDEC: accurate and efficient long read error correction. *Bioinformatics* 2014;**30**:3506–14.

13. Wu TD, Watanabe CK. GMAP: a genomic mapping and alignment program for mRNA and EST sequences. *Bioinformatics* 2005;**21**:1859–75.

14. Ou S, Su W, Liao Y *et al*. Benchmarking transposable element annotation methods for creation of a streamlined, comprehensive pipeline. *Genome Biol* 2019;**20**:275.

15. Slater GSC, Birney E. Automated generation of heuristics for biological sequence comparison. *BMC Bioinformatics* 2005;**6**:1–11.

16. Keller O, Kollmar M, Stanke M *et al.* A novel hybrid gene prediction method employing protein multiple sequence alignments. *Bioinformatics* 2011;**27**:757–63.

17. Majoros WH, Pertea M, Salzberg SL. TigrScan and GlimmerHMM: two open source *ab initio* eukaryotic gene-finders. *Bioinformatics* 2004;**20**:2878–9.

18. Jones P, Binns D, Chang H-Y *et al.* InterProScan 5: genome-scale protein function classification. *Bioinformatics* 2014;**30**:1236–40.

19. Conesa A, Götz S, García-Gómez JM *et al.* Blast2GO: a universal tool for annotation, visualization and analysis in functional genomics research. *Bioinformatics* 2005;**21**:3674–6.

20. Wang J, Luo J, Ma Y *et al.* Nuclear simple sequence repeat markers are superior to DNA barcodes for identification of closely related *Rhododendron* species on the same mountain. *J Syst Evol* 2019;**57**:278–86.

21. Yan L, Liu J, Möller M *et al.* DNA barcoding of *Rhododendron* (Ericaceae), the largest Chinese plant genus in biodiversity hotspots of the Himalaya-Hengduan Mountains. *Mol Ecol Resour* 2015;**15**:932–44.

22. Soza VL, Kriebel R, Ramage E *et al.* The symmetry spectrum in a hybridising, tropical group of rhododendrons. *New Phytol* 2022;**234**:1491–506.

23. Doyle JJ. A rapid DNA isolation procedure for small quantities of fresh leaf tissue. *Phytochem Bull* 1987;**19**:11–5.

24. Zha H, Milne RI, Sun H. Asymmetric hybridization in *Rhododendron agastum*: a hybrid taxon comprising mainly *F*_1_s in Yunnan, China. *Ann Bot* 2010;**105**:89–100

25. Xia X, Yang M, Li C *et al.* Spatiotemporal evolution of the global species diversity of *Rhododendron*. *Mol Biol Evol* 2021;**2021**:msab314.

26. Browning BL, Browning SR. Improving the accuracy and efficiency of identity-by-descent detection in population data. *Genetics* 2013;**194**:459–71.

27. Korunes KL, Samuk K. pixy: Unbiased estimation of nucleotide diversity and divergence in the presence of missing data. *Mol Ecol Resour* 2021;**21**:1359–68

28. Stamatakis A. RAxML version 8: a tool for phylogenetic analysis and post-analysis of large phylogenies. *Bioinformatics* 2014;**30**:1312–3.

29. Zhang C, Rabiee M, Sayyari E *et al.* ASTRAL-III: polynomial time species tree reconstruction from partially resolved gene trees. *BMC Bioinformatics* 2018;**19**:153.

30. Sayyari E, Whitfield JB, Mirarab S. DiscoVista: Interpretable visualizations of gene tree discordance. *Mol Phylogenet Evol* 2018;**122**:110–5.

31. Wick RR, Judd LM, Gorrie CL *et al.* Unicycler: Resolving bacterial genome assemblies from short and long sequencing reads. *PLOS Comput Biol* 2017;**13**:e1005595.

32. Koren S, Walenz BP, Berlin K *et al.* Canu: scalable and accurate long-read assembly via adaptive *k*-mer weighting and repeat separation. *Genome Res* 2017;**27**:722–36.

33. Yang Z. PAML 4: phylogenetic analysis by maximum likelihood. *Mol Biol Evol* 2007;**24**:1586–91.

34. Collinson ME, Crane PR. *Rhododendron* seeds from the Palaeocene of southern England. *Bot J Linn Soc* 1978;**76**:195–205.

35. Schwery O, Onstein RE, Bouchenak‐Khelladi Y *et al.* As old as the mountains: the radiations of the Ericaceae. *New Phytol* 2015;**207**:355–67.

36. Green RE, Krause J, Briggs AW *et al.* A draft sequence of the neandertal genome. *Science* 2010;**328**:710–22.

37. Malinsky M, Svardal H, Tyers AM *et al.* Whole-genome sequences of Malawi cichlids reveal multiple radiations interconnected by gene flow. *Nat Ecol Evol* 2018;**2**:1940–55.

38. Cai L, Xi Z, Lemmon EM *et al.* The perfect storm: gene tree estimation error, incomplete lineage sorting, and ancient gene flow explain the most recalcitrant ancient angiosperm clade, Malpighiales. *Syst Biol* 2021;**70**:491–507.
