# Supplementary material for: Pervasive hybridization during evolutionary radiation of Rhododendron subgenus Hymenanthes in mountains of southwest China
Source: Natl Sci Rev. 2022 Dec 2;9(12):nwac276. doi: 10.1093/nsr/nwac276 (PMC9844246; doi:10.1093/nsr/nwac276)
Supplement: nwac276_Supplemental_Files [file nwac276_supplemental_files.zip › Supplementary_Data-Tables.docx]

**P****ervasive hybridization during evolutionary radiation of *Rhododendron* subgenus *Hymenanthes* in mountains of southwest China**

Yazhen Ma^1,2^†, Xingxing Mao^1^†, Ji Wang^1^†, Lei Zhang^1^, Yuanzhong Jiang^1^, Yuying Geng^1^, Tao Ma^1^, Liming Cai^3^, Shuangquan Huang^4^, Pete Hollingsworth^5^, Kangshan Mao^1^, Minghui Kang^1^, Yiling Li^1^, Wenlu Yang^1^, Haolin Wu^1^, Yang Chen^1^, Charles C. Davis^3^,Nawal Shrestha^2^, Richard H. Ree^6^, Zhenxiang Xi^1^, Quanjun Hu^1^*, Richard I. Milne^5,7^*, Jianquan Liu^1,2^*

*Corresponding authors. Email: liujq@nwipb.cas.cn; r.milne@ed.ac.uk; huquanjun@scu.edu.cn.

Tables S1 to S9

Table S1. Genome sequencing data for *R. prattii*.

| Libraries | Insert size (bp) | Total data (G) | Sequence coverage (X) |
| --- | --- | --- | --- |
| Illumina reads | 350 | 75.28 | 111.86 |
| PacBio reads | 20,000 | 70.87 | 105.30 |
| 10X Genomics | 500-700 | 115.21 | 171.19 |
| Hi-C | 350 | 80.20 | 119.17 |
| Total | - | 261.36 | 388.35 |

Table S2. Summary of the Hi-C grouping result in each chromosome showing the number of scaffolds which were anchored and their total length in genomes of *R. prattii*.

| Chr^*^ | Scaffold number | Total length |
| --- | --- | --- |
| 1 | 102 | 66,739,168 |
| 2 | 98 | 65,797,546 |
| 3 | 117 | 63,457,898 |
| 4 | 89 | 54,363,032 |
| 5 | 92 | 52,586,643 |
| 6 | 73 | 52,504,019 |
| 7 | 70 | 51,783,352 |
| 8 | 86 | 49,415,980 |
| 9 | 78 | 49,256,243 |
| 10 | 87 | 45,324,500 |
| 11 | 47 | 42,935,356 |
| 12 | 51 | 40,014,900 |
| 13 | 52 | 35,291,825 |
| Total Sequences Clustered (Ratio %) | 1,042 (77.47) | 669,470,462 (99.46) |
| Total Sequences Ordered and Oriented (Ratio %) | 686 (65.83) | 631,086,395 (93.76) |

*The pseudo-chromosomes.

Table S3. Summary of *R. prattii* genome assembly.

| Assembly length (Mb) | Scaffold number | Scaffold N50 (Mb) | Scaffold N90 (Mb) | Contig number | Contig N50 (Kb) | Contig N90 (Kb) | N content (%) | GC cotent (%) |
| --- | --- | --- | --- | --- | --- | --- | --- | --- |
| 673.07 | 684 | 47.141 | 32.815 | 1,843 | 787.314 | 204.912 | 0.29 | 40.45 |

Table S4. Repeat abundance and composition in *R. prattii* genome.

| Class |  | Count | Bases | Percentage (%) |
| --- | --- | --- | --- | --- |
| LTR | Copia | 36,714 | 23,386,466 | 3.48% |
|  | Gypsy | 90,888 | 128,397,144 | 19.13% |
|  | unknown | 110,326 | 63,985,325 | 9.53% |
| TIR | CACTA | 45,603 | 12,774,002 | 1.90% |
|  | Mutator | 134,928 | 37,896,878 | 5.65% |
|  | PIF_Harbinger | 42,348 | 10,994,185 | 1.64% |
|  | Tc1_Mariner | 108,453 | 24,559,384 | 3.66% |
|  | hAT | 86,097 | 25,302,258 | 3.77% |
| nonLTR | LINE_element | 3,426 | 2,428,706 | 0.36% |
|  | unknown | 317 | 133,728 | 0.02% |
| nonTIR | helitron | 81,574 | 20,554,774 | 3.06% |
| repeat_region |  | 106,707 | 33,873,985 | 5.05% |
| Total |  | 847,381 | 384,286,835 | 57.26% |

Table S5. The features of the predicted genes of *R. prattii* genome.

| Total Genes | Average gene length (bp) | Average CDS length (bp) | Average exon length (bp) | Average intron length (bp) | Average exons per gene |
| --- | --- | --- | --- | --- | --- |
| 37,092 | 6,002.34 | 1,230.65 | 253.97 | 1060.30 | 4.85 |

Table S6. The annotated genes of *R. prattii* genomes by different databases.

|  | | Database | Total gene number | Percentage |
| --- | --- | --- | --- | --- |
| Total | |  | 37,092 |  |
| Annotated | | InterPro | 35,000 | 94.36% |
|  | | GO | 30,010 | 80.91% |
|  | | KEGG | 9,258 | 24.96% |
|  | | Swissprot | 26,151 | 70.50% |
|  | | NR | 36,056 | 97.21% |
| Unannotated |  | | 569 | 1.53% |
| Total annotated | | | 36,523 | 96.12% |

Table S7. Comparisons of genome assemblies and annotations among *R. prattii* and other published *Rhododendron* genomes.

| Species | Subgenus | Heterozygosity (%) | Assembly size (Mb) | Number of scaffolds | Scafold N50 (Mb) | Complete BUSCOs of assembly (%) | Repeat abundance | Number of protein-coding genes | Complete BUSCOs of annotation (%) |
| --- | --- | --- | --- | --- | --- | --- | --- | --- | --- |
| *R. prattii* | *Hymenanthes* | 1.2 | 673.07 | 684 | 47.14 | 97.3 | 57.26% | 37,092 | 91.8 |
| *R. delavayi* [1] | *Hymenanthes* | 0.9 | 695.09 | 193,091 | 0.64 | 96.0 | 51.77% | 32,938 | 86.2 |
| *R. williamsianum* [2] | *Hymenanthes* | NA | 532.29 | 10,290 | 29.01 | 94.2 | 58.80% | 23,365 | 76.9 |
| *R. griersonianum* [3] | *Hymenanthes* | 0.18 | 674.98 | 47 | 52.93 | 98.2 | 57.00% | 38,146 | 84.7 |
| *R. henanense* [4] | *Hymenanthes* | 0.72 | 634.28 | 300 | 50.18 | 98.2 | 65.76% | 31,098 | 94.1 |
| *R. simsii* [5] | *Tsutsusi* | 1.78 | 528.64 | 552 | 36.35 | 97.7 | 47.48% | 32,999 | 90.4 |
| *R. ovatum* [6] | *Tsutsusi* | 1.55 | 549.71 | 14 | 41.12 | 98.3 | 44.70% | 41,292 | 92.5 |
| *R. ripense* [7] | *Tsutsusi* | NA | 506.73 | 78 | 37.02 | 97.5 | 51.40% | 34,606 | 80.8 |

Table S8. Summary statistics of whole-genome resequencing data for 277 individuals.

| Analysis ID | Voucher ID | Species | Subgenus | Longitude (°E) | Latitude (°N) | Average  depth | Mapping  coverage |
| --- | --- | --- | --- | --- | --- | --- | --- |
| RH058 | LJQMXX201644 | *R. adenogynum* | *Hymenanthes* | 100.21 | 27.16 | 42.26 | 0.91 |
| RH042 | 19698334 | *R. adenopodum* | *Hymenanthes* | 108.73 | 31.88 | 35.46 | 0.89 |
| RH133 | 19300433 | *R. adenosum* | *Hymenanthes* | 100.86 | 28.38 | 38.43 | 0.90 |
| RH171 | ML170263 | *R. aganniphum* var. *schizopeplum* | *Hymenanthes* | 89.04 | 27.57 | 37.07 | 0.89 |
| RH237 | Ljq_Zl_038 | *R. agastum* | *Hymenanthes* | 105.83 | 27.22 | 42.83 | 0.92 |
| RH108 | MW123 | *R. anthosphaerum* | *Hymenanthes* | 98.46 | 27.84 | 37.30 | 0.89 |
| RH234 | 19710038 | *R. anwheiense* | *Hymenanthes* | 115.40 | 30.80 | 40.46 | 0.89 |
| RH258 | Mao_NPL_74 | *R. arboreum* | *Hymenanthes* | 85.33 | 28.08 | 47.26 | 0.89 |
| RH152 | LJL_2017_20 | *R. argyrophyllum* | *Hymenanthes* | 102.65 | 30.41 | 41.46 | 0.91 |
| RH203 | MW131 | *R. arizelum* | *Hymenanthes* | 98.46 | 27.84 | 29.70 | 0.88 |
| RH312 | LJQ_ZL_2015_06 | *R. asterochnoum* | *Hymenanthes* | 103.77 | 31.57 | 33.26 | 0.89 |
| RH313 | ML2019110 | *R. asterochnoum* | *Hymenanthes* | 103.03 | 30.91 | 37.92 | 0.89 |
| RH119 | 2017_CBS_1 | *R. aureum* | *Hymenanthes* | 128.07 | 42.04 | 41.45 | 0.82 |
| RH008 | 19160027 | *R. auriculatum* | *Hymenanthes* | 108.40 | 30.25 | 31.28 | 0.88 |
| RH093 | ML170325 | *R. auriculatum* | *Hymenanthes* | 110.12 | 31.45 | 39.35 | 0.88 |
| RH153 | GZW_2017_07 | *R. balangense* | *Hymenanthes* | 103.20 | 31.05 | 36.72 | 0.90 |
| RH154 | LJQ_ZL_2017_07 | *R. balangense* | *Hymenanthes* | 104.16 | 32.91 | 32.75 | 0.92 |
| RH017 | 19720856 | *R. barbatum* | *Hymenanthes* | 87.17 | 27.75 | 29.84 | 0.88 |
| RH259 | Mao_NPL_61 | *R. barbatum* | *Hymenanthes* | 85.34 | 28.12 | 42.87 | 0.90 |
| RH239 | 19291021 | *R. beanianum* | *Hymenanthes* | 97.40 | 28.10 | 42.05 | 0.89 |
| RH080 | ML170177 | *R. beesianum* | *Hymenanthes* | 95.70 | 29.79 | 37.55 | 0.90 |
| RH130 | 19568214 | *R. brachycarpum* | *Hymenanthes* | 139.40 | 36.80 | 41.78 | 0.82 |
| RH185 | ML17115 | *R. brevinerve* | *Hymenanthes* | 109.95 | 25.60 | 41.60 | 0.88 |
| RH290 | MYJ024-A | *R. brevinerve* | *Hymenanthes* | 110.24 | 24.17 | 39.57 | 0.88 |
| RH195 | MW064 | *R. bureavii* | *Hymenanthes* | 100.11 | 26.25 | 37.37 | 0.92 |
| RH310 | MMW006 | *R. calophytum* | *Hymenanthes* | 103.33 | 29.54 | 44.06 | 0.89 |
| RH311 | ML2019126 | *R. calophytum* | *Hymenanthes* | 102.31 | 29.88 | 36.32 | 0.89 |
| RH315 | cxn201944 | *R. calophytum* var. *openshawianum* | *Hymenanthes* | 102.95 | 29.66 | 27.08 | 0.87 |
| RH308 | ML2019158 | *R. calophytum* var. *pauciflorum* | *Hymenanthes* | 107.18 | 29.02 | 38.72 | 0.89 |
| RH309 | ML2019158 | *R. calophytum* var. *pauciflorum* | *Hymenanthes* | 107.18 | 29.02 | 35.39 | 0.89 |
| RH111 | Mao_NPL_71 | *R. campanulatum* | *Hymenanthes* | 85.33 | 28.08 | 39.23 | 0.89 |
| RH167 | ML170225 | *R. campylocarpum* | *Hymenanthes* | 95.00 | 29.47 | 36.43 | 0.91 |
| RH011 | 19340114 | *R. catawbiense* | *Hymenanthes* | 81.88 | 35.89 | 28.94 | 0.82 |
| RH013 | 19521068 | *R. caucasicum* | *Hymenanthes* | 41.21 | 41.00 | 35.75 | 0.81 |
| RH082 | ML170207 | *R. cerasinum* | *Hymenanthes* | 95.00 | 29.47 | 40.93 | 0.90 |
| RH182 | MSC1713 | *R. chihsinianum* | *Hymenanthes* | 111.01 | 26.37 | 36.82 | 0.88 |
| RH037 | 19231045 | *R. coriaceum* | *Hymenanthes* | 98.80 | 28.23 | 33.08 | 0.89 |
| RH123 | 19698489 | *R. coryanum* | *Hymenanthes* | 94.40 | 29.60 | 38.68 | 0.89 |
| RH043 | 19698494 | *R. crinigerum* | *Hymenanthes* | 99.26 | 27.44 | 28.38 | 0.89 |
| RH198 | MW096 | *R. cyanocarpum* | *Hymenanthes* | 100.03 | 25.87 | 36.01 | 0.88 |
| RH033 | LJQ_ZL_2015_10 | *R. davidii* | *Hymenanthes* | 103.58 | 31.13 | 37.96 | 0.90 |
| RH113 | SWZML2017108 | *R. decorum* | *Hymenanthes* | 102.35 | 28.78 | 38.90 | 0.90 |
| RH217 | Ljq_Zl_043 | *R. decorum* | *Hymenanthes* | 105.83 | 27.22 | 52.31 | 0.91 |
| RH246 | ML17058 | *R. decorum* | *Hymenanthes* | 102.96 | 30.54 | 36.14 | 0.90 |
| RH265 | 19761903 | *R. degronianum* | *Hymenanthes* | 139.62 | 36.75 | 38.25 | 0.83 |
| RH099 | ML17085 | *R. delavayi* | *Hymenanthes* | 105.47 | 25.39 | 39.67 | 0.88 |
| RH141 | Ljq_Zl_031 | *R. delavayi* | *Hymenanthes* | 104.62 | 26.12 | 30.74 | 0.86 |
| RH200 | MW107 | *R. delavayi* | *Hymenanthes* | 100.03 | 25.87 | 37.70 | 0.87 |
| RH209 | SWZML2017115 | *R. denudatum* | *Hymenanthes* | 102.35 | 28.78 | 31.25 | 0.89 |
| RH260 | SWZML201746 | *R. detersile* | *Hymenanthes* | 108.68 | 31.60 | 36.56 | 0.91 |
| RH019 | 19730296B | *R. dichroanthum* | *Hymenanthes* | 98.67 | 25.67 | 31.74 | 0.87 |
| RH174 | ML170320 | *R. discolor* | *Hymenanthes* | 110.26 | 30.80 | 35.95 | 0.90 |
| RH241 | ML170221 | *R. exasperatum* | *Hymenanthes* | 95.09 | 29.40 | 44.24 | 0.90 |
| RH164 | GZW_2017_35 | *R. faberi* | *Hymenanthes* | 104.00 | 31.69 | 32.49 | 0.93 |
| RH255 | MW089 | *R. facetum* | *Hymenanthes* | 100.02 | 25.85 | 41.54 | 0.89 |
| RH268 | MSC1701 | *R. faithae* | *Hymenanthes* | 110.54 | 22.37 | 43.15 | 0.90 |
| RH007 | 19141002 | *R. falconeri* | *Hymenanthes* | 88.27 | 27.03 | 29.69 | 0.88 |
| RH129 | 19470106 | *R. faucium* | *Hymenanthes* | 95.53 | 30.13 | 44.27 | 0.90 |
| RH232 | MW122 | *R. floccigerum* | *Hymenanthes* | 98.46 | 27.84 | 41.02 | 0.89 |
| RH056 | Ljq_Zl_029 | *R. floribundum* | *Hymenanthes* | 104.62 | 26.12 | 37.81 | 0.89 |
| RH144 | Ljq_Zl_057 | *R. floribundum* | *Hymenanthes* | 105.83 | 27.22 | 41.78 | 0.89 |
| RH083 | ML170208 | *R. forrestii* | *Hymenanthes* | 95.00 | 29.47 | 35.64 | 0.91 |
| RH184 | ML17111 | *R. fortunei* | *Hymenanthes* | 109.95 | 25.60 | 32.59 | 0.88 |
| RH030 | 19371010 | *R. fulgens* | *Hymenanthes* | 88.90 | 27.50 | 40.53 | 0.88 |
| RH127 | 19180010 | *R. fulvum* | *Hymenanthes* | 98.80 | 28.23 | 34.83 | 0.90 |
| RH222 | GZW_2017_08 | *R. galactinum* | *Hymenanthes* | 103.60 | 31.48 | 40.32 | 0.89 |
| RH115 | 2018R002 | *R. glanduliferum* | *Hymenanthes* | 103.90 | 27.45 | 38.84 | 0.90 |
| RH168 | ML170205 | *R. glischrum* | *Hymenanthes* | 95.00 | 29.47 | 40.42 | 0.89 |
| RH104 | MW034 | *R. gonggashanense* | *Hymenanthes* | 101.54 | 29.47 | 38.79 | 0.90 |
| RH191 | MW026 | *R. gonggashanense* | *Hymenanthes* | 101.77 | 29.54 | 36.16 | 0.90 |
| RH230 | 19698606 | *R. grande* | *Hymenanthes* | 95.30 | 29.30 | 45.49 | 0.92 |
| RH134 | 19320271 | *R. griersonianum* | *Hymenanthes* | 98.46 | 25.33 | 42.10 | 0.86 |
| RH236 | 2015_GER_14 | *R. habrotrichum* | *Hymenanthes* | 98.50 | 25.50 | 41.72 | 0.89 |
| RH060 | LJQMXX201622 | *R. haematodes* | *Hymenanthes* | 100.09 | 25.68 | 42.67 | 0.89 |
| RH188 | MSC1726 | *R. haofui* | *Hymenanthes* | 114.16 | 26.51 | 34.87 | 0.87 |
| RH261 | SWZML2017102 | *R. hemsleyanum* | *Hymenanthes* | 103.36 | 29.56 | 33.36 | 0.89 |
| RH086 | ML170294 | *R. hirtipes* | *Hymenanthes* | 94.58 | 29.56 | 37.90 | 0.90 |
| RH041 | 19902809 | *R. hodgsonii* | *Hymenanthes* | 91.00 | 27.40 | 44.12 | 0.89 |
| RH116 | SWZML2017105 | *R. huanum* | *Hymenanthes* | 103.42 | 29.05 | 40.58 | 0.90 |
| RH053 | Ljq_Zl_012 | *R. hunnewellianum* | *Hymenanthes* | 103.40 | 30.68 | 29.86 | 0.88 |
| RH242 | ML170222 | *R. hylaeum* | *Hymenanthes* | 95.09 | 29.40 | 37.89 | 0.91 |
| RH131 | 19568422 | *R. hyperythrum* | *Hymenanthes* | 120.96 | 23.47 | 39.77 | 0.87 |
| RH112 | SWZML201729 | *R. hypoglaucum* | *Hymenanthes* | 108.68 | 31.83 | 45.00 | 0.90 |
| RH016 | 19698662 | *R. insigne* | *Hymenanthes* | 102.90 | 29.70 | 32.30 | 0.87 |
| RH190 | MW093 | *R. irroratum* | *Hymenanthes* | 100.10 | 25.85 | 34.81 | 0.89 |
| RH189 | MSC1734 | *R. jingangshanicum* | *Hymenanthes* | 114.08 | 26.44 | 41.17 | 0.88 |
| RH314 | MYJ029 | *R. jingangshanicum* | *Hymenanthes* | 114.16 | 26.56 | 24.74 | 0.85 |
| RH049 | 2015_GER_20 | *R. kesangiae* | *Hymenanthes* | 89.70 | 27.50 | 37.26 | 0.88 |
| RH055 | LJQMXX201610 | *R. lacteum* | *Hymenanthes* | 100.08 | 25.68 | 38.99 | 0.90 |
| RH025 | ML170267 | *R. lanatum* | *Hymenanthes* | 89.04 | 27.57 | 32.58 | 0.84 |
| RH010 | 19291008 | *R. lanigerum* | *Hymenanthes* | 85.30 | 28.90 | 30.41 | 0.86 |
| RH160 | GZW_2017_32 | *R. longesquamatum* | *Hymenanthes* | 104.00 | 31.69 | 32.89 | 0.93 |
| RH110 | SWZML201790 | *R. longipes* var. *chienianum* | *Hymenanthes* | 107.19 | 29.02 | 44.80 | 0.90 |
| RH034 | Liu_2015_11_16 | *R. lulangense* | *Hymenanthes* | 94.25 | 29.59 | 38.99 | 0.90 |
| RH031 | 19698707 | *R. macabeanum* | *Hymenanthes* | 95.00 | 26.00 | 38.37 | 0.89 |
| RH264 | 19734184 | *R. macrophyllum* | *Hymenanthes* | -123.25 | 49.25 | 39.04 | 0.83 |
| RH095 | ML170332 | *R. maculiferum* | *Hymenanthes* | 110.47 | 31.53 | 37.56 | 0.90 |
| RH100 | ML17088 | *R. magniflorum* | *Hymenanthes* | 105.46 | 25.38 | 39.60 | 0.90 |
| RH135 | 19380170 | *R. makinoi* | *Hymenanthes* | 137.30 | 35.00 | 31.22 | 0.82 |
| RH102 | ML17119 | *R. maoerense* | *Hymenanthes* | 110.46 | 25.91 | 41.39 | 0.89 |
| RH020 | 19850601 | *R. maximum* | *Hymenanthes* | 87.03 | 35.20 | 38.05 | 0.78 |
| RH024 | 2018_LiuUS_01 | *R. maximum* | *Hymenanthes* | -80.31 | 38.86 | 34.10 | 0.76 |
| RH048 | 19980009 | *R. meddianum* | *Hymenanthes* | 98.97 | 25.50 | 37.00 | 0.88 |
| RH139 | 2015_GER_24 | *R. microgynum* | *Hymenanthes* | 99.09 | 28.40 | 40.28 | 0.91 |
| RH266 | 19773076 | *R. morii* | *Hymenanthes* | 120.96 | 23.47 | 40.85 | 0.89 |
| RH219 | LJQMXX201615 | *R. neriiflorum* | *Hymenanthes* | 100.10 | 25.69 | 46.24 | 0.90 |
| RH136 | 19701316 | *R. niveum* | *Hymenanthes* | 88.70 | 27.80 | 44.55 | 0.87 |
| RH051 | 2015_GER_82 | *R. ochraceum* | *Hymenanthes* | 103.10 | 29.20 | 41.85 | 0.89 |
| RH092 | ML17069 | *R. orbiculare* | *Hymenanthes* | 102.52 | 30.38 | 35.55 | 0.89 |
| RH186 | ML17120 | *R. orbiculare* ssp. *oblongum* | *Hymenanthes* | 110.46 | 25.91 | 43.62 | 0.89 |
| RH032 | LJQ_ZL_2015_01 | *R. oreodoxa* | *Hymenanthes* | 103.77 | 31.57 | 32.10 | 0.89 |
| RH096 | ML17035 | *R. oreodoxa* | *Hymenanthes* | 102.98 | 30.88 | 42.10 | 0.90 |
| RH094 | ML170331 | *R. oreodoxa* var. *fargesii* | *Hymenanthes* | 110.45 | 31.53 | 44.95 | 0.89 |
| RH251 | MSC1717 | *R. pachyphyllum* | *Hymenanthes* | 111.01 | 26.37 | 44.25 | 0.90 |
| RH176 | ML17052 | *R. pachytrichum* | *Hymenanthes* | 102.96 | 30.54 | 36.57 | 0.91 |
| RH126 | Liu_2015_11_06 | *R. phaeochrysum* | *Hymenanthes* | 94.25 | 29.59 | 40.46 | 0.91 |
| RH172 | ML170292 | *R. phaeochrysum* | *Hymenanthes* | 94.58 | 29.56 | 35.19 | 0.90 |
| RH068 | LJL_2017_09 | *R. pingianum* | *Hymenanthes* | 103.09 | 28.72 | 36.04 | 0.90 |
| RH054 | Ljq_Zl_086 | *R. platypodum* | *Hymenanthes* | 107.13 | 29.05 | 41.02 | 0.89 |
| RH999 | 19773079 | *R. ponticum* | *Hymenanthes* | 39.64 | 40.72 | 34.94 | 0.79 |
| RH038 | 19240357 | *R. praevernum* | *Hymenanthes* | 110.26 | 30.81 | 37.41 | 0.89 |
| RH306 | my117 | *R. praevernum* | *Hymenanthes* | 110.26 | 30.81 | 28.12 | 0.87 |
| RH992 | 2017_LJQ_JDS01 | *R. prattii* | *Hymenanthes* | 103.75 | 31.52 | 27.94 | 0.99 |
| RH035 | Liu_2015_11_18 | *R. principis* | *Hymenanthes* | 94.25 | 29.59 | 37.71 | 0.89 |
| RH201 | MW120 | *R. protistum* | *Hymenanthes* | 98.59 | 27.79 | 38.33 | 0.88 |
| RH074 | GZW_2017_23 | *R. przewalskii* | *Hymenanthes* | 102.74 | 30.81 | 31.68 | 0.91 |
| RH155 | LJQ_ZL_2017_08 | *R. przewalskii* | *Hymenanthes* | 104.16 | 32.91 | 40.06 | 0.91 |
| RH040 | 19810864 | *R. pseudochrysanthum* | *Hymenanthes* | 120.81 | 23.52 | 36.05 | 0.87 |
| RH044 | 19764021 | *R. pudorosum* | *Hymenanthes* | 92.91 | 28.49 | 46.36 | 0.90 |
| RH163 | ML170190 | *R. ramsdenianum* | *Hymenanthes* | 95.52 | 29.70 | 36.76 | 0.89 |
| RH194 | MW059 | *R. rex* ssp. *fictolacteum* | *Hymenanthes* | 99.74 | 27.08 | 40.54 | 0.90 |
| RH199 | MW105 | *R. rex* ssp. *fictolacteum* | *Hymenanthes* | 99.99 | 25.87 | 30.87 | 0.89 |
| RH158 | LJL_2017_01 | *R. ririei* | *Hymenanthes* | 103.09 | 28.72 | 35.79 | 0.89 |
| RH061 | LJQMXX201635 | *R. roxieanum* | *Hymenanthes* | 99.60 | 26.93 | 45.01 | 0.92 |
| RH073 | GZW_2017_22 | *R. rufum* | *Hymenanthes* | 102.74 | 30.81 | 35.57 | 0.91 |
| RH151 | MXXLJQ043 | *R. selense* | *Hymenanthes* | 99.63 | 27.80 | 37.42 | 0.93 |
| RH052 | 2015_GER_90 | *R. sherriffii* | *Hymenanthes* | 95.50 | 30.40 | 41.44 | 0.90 |
| RH076 | ML170189 | *R. sidereum* | *Hymenanthes* | 95.52 | 29.70 | 37.25 | 0.89 |
| RH159 | GZW_2017_27 | *R. sikangense* | *Hymenanthes* | 102.39 | 29.92 | 36.77 | 0.91 |
| RH228 | LZQ_2015_07 | *R. simiarum* | *Hymenanthes* | 112.92 | 24.94 | 36.01 | 0.86 |
| RH181 | ML17099 | *R. simiarum* var. *versicolor* | *Hymenanthes* | 110.11 | 23.97 | 41.47 | 0.85 |
| RH047 | 19960615 | *R. sinofalconeri* | *Hymenanthes* | 104.50 | 27.83 | 38.31 | 0.89 |
| RH014 | 19623832 | *R. smirnowii* | *Hymenanthes* | 41.60 | 41.30 | 33.29 | 0.79 |
| RH088 | ML170312 | *R. souliei* | *Hymenanthes* | 101.86 | 30.01 | 37.48 | 0.89 |
| RH097 | ML17053 | *R. strigillosum* | *Hymenanthes* | 102.96 | 30.54 | 38.78 | 0.92 |
| RH175 | ML170323 | *R. sutchuenense* | *Hymenanthes* | 110.14 | 31.45 | 38.33 | 0.89 |
| RH307 | MY130 | *R. sutchuenense* | *Hymenanthes* | 110.16 | 31.30 | 38.46 | 0.89 |
| RH149 | MXXLJQ009 | *R. taliense* | *Hymenanthes* | 100.10 | 25.66 | 38.99 | 0.90 |
| RH125 | Liu_2015_11_01 | *R. tanastylum* var. *lingzhiense* | *Hymenanthes* | 94.25 | 29.59 | 38.28 | 0.90 |
| RH039 | 19370196 | *R. thomsonii* | *Hymenanthes* | 91.82 | 27.92 | 47.08 | 0.90 |
| RH050 | 2015_GER_39 | *R. tsariense* | *Hymenanthes* | 94.60 | 29.30 | 39.17 | 0.86 |
| RH252 | 19568672 | *R. ungernii* | *Hymenanthes* | 41.90 | 41.50 | 48.72 | 0.83 |
| RH193 | MW049 | *R. uvariifolium* | *Hymenanthes* | 99.61 | 26.95 | 37.28 | 0.90 |
| RH245 | 19370170 | *R. venator* | *Hymenanthes* | 94.00 | 29.26 | 40.30 | 0.89 |
| RH078 | ML170129 | *R. vernicosum* | *Hymenanthes* | 101.11 | 31.88 | 38.07 | 0.89 |
| RH103 | MW018 | *R. verruciferum* | *Hymenanthes* | 101.22 | 30.89 | 38.84 | 0.90 |
| RH036 | 19734094 | *R. wallichii* | *Hymenanthes* | 91.00 | 28.30 | 42.13 | 0.89 |
| RH147 | MXXLJQ071 | *R. wardii* | *Hymenanthes* | 99.15 | 28.31 | 33.93 | 0.91 |
| RH165 | GZW_2017_38 | *R. wasonii* var. *wenchuanense* | *Hymenanthes* | 104.00 | 31.69 | 42.44 | 0.93 |
| RH128 | 19320138 | *R. williamsianum* | *Hymenanthes* | 103.40 | 29.50 | 35.72 | 0.89 |
| RH173 | ML17073 | *R. wiltonii* | *Hymenanthes* | 102.56 | 30.43 | 37.10 | 0.93 |
| RH064 | GZW_2017_02 | *R. wolongense* | *Hymenanthes* | 103.31 | 31.10 | 40.74 | 0.88 |
| RH253 | MSC1736 | *R. xiaoxidongense* | *Hymenanthes* | 114.06 | 26.43 | 31.66 | 0.87 |
| RH015 | 19660141 | *R. yakushimanum* | *Hymenanthes* | 130.52 | 30.31 | 32.66 | 0.79 |
| RH301 | CXN201942 | *R. molle* | *Pentanthera* | 103.08 | 29.64 | 33.34 | 0.68 |
| RH302 | MYJ042 | *R. molle* | *Pentanthera* | 102.74 | 25.14 | 29.61 | 0.67 |
| RH002 | 19741229 | *R. molle ssp. japonicum* | *Pentanthera* | 140.20 | 38.24 | 33.58 | 0.68 |
| RH161 | GZW_2017_09 | *R. ambiguum* | *Rhododendron* | 103.20 | 31.05 | 37.32 | 0.79 |
| RH178 | ML17074 | *R. amesiae* | *Rhododendron* | 102.56 | 30.43 | 40.42 | 0.79 |
| RH197 | Mao_NPL_33 | *R. anthopogon* | *Rhododendron* | 85.63 | 28.21 | 47.68 | 0.74 |
| RH075 | GZW_2017_24 | *R. anthopogonoides* | *Rhododendron* | 102.74 | 30.81 | 41.39 | 0.72 |
| RH162 | GZW_2017_10 | *R. augustinii* | *Rhododendron* | 103.20 | 31.05 | 36.88 | 0.78 |
| RH109 | SWZML201780 | *R. brachypodum* | *Rhododendron* | 107.25 | 28.98 | 30.05 | 0.71 |
| RH079 | ML170175 | *R. calostrotum* | *Rhododendron* | 95.70 | 29.79 | 33.40 | 0.73 |
| RH187 | MW016 | *R. capitatum* | *Rhododendron* | 101.35 | 30.73 | 31.70 | 0.76 |
| RH140 | Ljq_Zl_088 | *R. changii* | *Rhododendron* | 107.13 | 29.05 | 39.62 | 0.73 |
| RH169 | ML170210 | *R. charitopes* ssp. *tsangpoense* | *Rhododendron* | 95.00 | 29.47 | 36.77 | 0.74 |
| RH120 | 2018R009 | *R. ciliicalyx* | *Rhododendron* | 102.73 | 25.13 | 39.88 | 0.73 |
| RH087 | ML170306 | *R. complexum* | *Rhododendron* | 98.49 | 29.70 | 39.16 | 0.74 |
| RH069 | GZW_2017_31 | *R. concinnum* | *Rhododendron* | 104.00 | 31.69 | 39.94 | 0.78 |
| RH118 | SWZML2017122 | *R. cuneatum* | *Rhododendron* | 102.40 | 28.77 | 34.18 | 0.77 |
| RH157 | LJQ_ZL_2017_14 | *R. dauricum* | *Rhododendron* | 128.07 | 42.02 | 40.70 | 0.75 |
| RH218 | Ljq_Zl_050 | *R. davidsonianum* | *Rhododendron* | 105.83 | 27.22 | 33.24 | 0.80 |
| RH257 | MW118 | *R. dendricola* | *Rhododendron* | 98.64 | 27.77 | 37.74 | 0.73 |
| RH098 | ML17054 | *R. dendrocharis* | *Rhododendron* | 102.96 | 30.53 | 33.22 | 0.73 |
| RH059 | LJQMXX201612 | *R. edgeworthii* | *Rhododendron* | 100.11 | 25.70 | 38.90 | 0.71 |
| RH085 | ML170212 | *R. hypenanthum* | *Rhododendron* | 95.00 | 29.47 | 33.34 | 0.79 |
| RH240 | ML170193 | *R. keysii* | *Rhododendron* | 95.55 | 29.70 | 35.23 | 0.81 |
| RH272 | MSC1729 | *R. kiangsiense* | *Rhododendron* | 114.16 | 26.51 | 38.77 | 0.70 |
| RH166 | ML170176 | *R. kongboense* | *Rhododendron* | 95.70 | 29.79 | 35.91 | 0.77 |
| RH211 | 2017_CBS_3 | *R. lapponicum* | *Rhododendron* | 128.07 | 42.04 | 36.77 | 0.69 |
| RH205 | Mao_NPL_10 | *R. lepidotum* | *Rhododendron* | 85.57 | 28.22 | 41.97 | 0.73 |
| RH170 | ML170218 | *R. leptocarpum* | *Rhododendron* | 95.09 | 29.40 | 39.00 | 0.73 |
| RH138 | Ljq_Zl_023 | *R. liliiflorum* | *Rhododendron* | 104.39 | 25.72 | 37.69 | 0.75 |
| RH183 | MSC1716 | *R. liliiflorum* | *Rhododendron* | 111.01 | 26.37 | 38.18 | 0.75 |
| RH269 | LJL_2017_18 | *R. lutescens* | *Rhododendron* | 102.65 | 30.41 | 37.65 | 0.73 |
| RH063 | MXXLJQ007 | *R. maddenii* ssp. *crassum* | *Rhododendron* | 100.14 | 25.65 | 36.37 | 0.79 |
| RH202 | MW125 | *R. megeratum* | *Rhododendron* | 98.46 | 27.84 | 37.26 | 0.72 |
| RH223 | ML170206 | *R. mekongense* | *Rhododendron* | 95.00 | 29.47 | 37.71 | 0.74 |
| RH117 | SWZML2017117 | *R. mianningense* | *Rhododendron* | 102.40 | 28.77 | 33.91 | 0.77 |
| RH207 | MT1702 | *R. micranthum* | *Rhododendron* | 104.76 | 32.58 | 36.48 | 0.72 |
| RH105 | MW041 | *R. minyaense* | *Rhododendron* | 100.30 | 29.56 | 41.91 | 0.73 |
| RH067 | LJL_2017_08 | *R. moupinense* | *Rhododendron* | 103.09 | 28.72 | 35.26 | 0.70 |
| RH070 | GZW_2017_15 | *R. nitidulum* | *Rhododendron* | 102.37 | 31.00 | 42.94 | 0.75 |
| RH224 | ML170251 | *R. nivale* | *Rhododendron* | 88.98 | 27.81 | 38.79 | 0.74 |
| RH210 | chen_2017_75 | *R. nuttallii* | *Rhododendron* | 95.15 | 29.19 | 33.39 | 0.68 |
| RH146 | MXXLJQ049 | *R. oreotrephes* | *Rhododendron* | 99.63 | 27.80 | 45.12 | 0.79 |
| RH148 | MXXLJQ003 | *R. pachypodum* | *Rhododendron* | 100.14 | 25.65 | 36.28 | 0.73 |
| RH090 | ML170259 | *R. pendulum* | *Rhododendron* | 88.98 | 27.81 | 39.92 | 0.72 |
| RH072 | GZW_2017_20 | *R. polycladum* | *Rhododendron* | 102.37 | 31.00 | 42.18 | 0.73 |
| RH005 | LJQ_ZL_2015_08 | *R. polylepis* | *Rhododendron* | 103.58 | 31.13 | 29.86 | 0.69 |
| RH124 | LJQ_ZL_2015_08 | *R. polylepis* | *Rhododendron* | 103.58 | 31.13 | 36.87 | 0.75 |
| RH215 | ML170271 | *R. primuliflorum* | *Rhododendron* | 92.54 | 28.60 | 38.42 | 0.71 |
| RH196 | MW065 | *R. racemosum* | *Rhododendron* | 100.16 | 26.11 | 38.33 | 0.73 |
| RH106 | MW044 | *R. radendum* | *Rhododendron* | 99.76 | 28.95 | 39.66 | 0.73 |
| RH220 | MXXLJQ005 | *R. rigidum* | *Rhododendron* | 100.14 | 25.65 | 40.90 | 0.80 |
| RH213 | 2018R008 | *R. roseatum* | *Rhododendron* | 102.73 | 25.13 | 40.10 | 0.72 |
| RH062 | LJQMXX201649 | *R. rubiginosum* | *Rhododendron* | 100.10 | 26.38 | 37.93 | 0.79 |
| RH256 | MW090 | *R. rubiginosum* | *Rhododendron* | 100.02 | 25.85 | 38.42 | 0.78 |
| RH221 | MXXLJQ053 | *R. rupicola* var. *chryseum* | *Rhododendron* | 99.63 | 27.80 | 43.11 | 0.76 |
| RH077 | GZW_2017_36 | *R. sargentianum* | *Rhododendron* | 104.00 | 31.69 | 32.50 | 0.74 |
| RH262 | Ljq_Zl_047 | *R. scabrifolium* | *Rhododendron* | 105.83 | 27.22 | 34.99 | 0.76 |
| RH089 | ML170249 | *R. setosum* | *Rhododendron* | 88.98 | 27.81 | 40.26 | 0.71 |
| RH206 | Mao_NPL_08 | *R. setosum* | *Rhododendron* | 85.57 | 28.22 | 41.80 | 0.72 |
| RH045 | Ljq_Zl_021 | *R. siderophyllum* | *Rhododendron* | 104.39 | 25.72 | 28.59 | 0.77 |
| RH214 | 2018R013 | *R. siderophyllum* | *Rhododendron* | 102.62 | 25.07 | 46.74 | 0.76 |
| RH114 | 2018R001 | *R. spiciferum* | *Rhododendron* | 102.75 | 25.14 | 34.59 | 0.73 |
| RH226 | ML17079 | *R. spinuliferum* | *Rhododendron* | 101.95 | 27.10 | 37.89 | 0.77 |
| RH121 | 2018R025 | *R. sulfureum* | *Rhododendron* | 100.14 | 25.64 | 37.81 | 0.71 |
| RH254 | MW022 | *R. tatsienense* | *Rhododendron* | 105.34 | 29.59 | 35.30 | 0.74 |
| RH122 | 2017_ZL_CBS02 | *R. tomentosum* | *Rhododendron* | 128.44 | 42.03 | 45.98 | 0.73 |
| RH066 | LJQ_ZL_2017_12 | *R. trichostomum* | *Rhododendron* | 104.16 | 32.91 | 36.63 | 0.70 |
| RH006 | Liu_2015_11_17 | *R. triflorum* | *Rhododendron* | 94.25 | 29.59 | 31.59 | 0.68 |
| RH244 | ML170289 | *R. triflorum* | *Rhododendron* | 94.56 | 29.56 | 43.52 | 0.75 |
| RH238 | MXXLJQ004 | *R. virgatum* | *Rhododendron* | 100.14 | 25.65 | 41.71 | 0.74 |
| RH216 | ML170311 | *R. websterianum* | *Rhododendron* | 99.55 | 30.30 | 39.82 | 0.70 |
| RH243 | ML170258 | *R. xanthocodon* | *Rhododendron* | 88.98 | 27.81 | 40.45 | 0.80 |
| RH150 | MXXLJQ024 | *R. yunnanense* | *Rhododendron* | 100.20 | 27.02 | 31.59 | 0.84 |
| RH001 | 19699487 | *R. camtschaticum* | *Therorhodion* | 148.00 | 45.10 | 38.37 | 0.49 |
| RH990 | 2017_ZL_CBS01 | *R. redowskianum* | *Therorhodion* | 128.07 | 42.04 | 45.55 | 0.50 |
| RH107 | MW131_1 | *R. atrovirens* | *Tsutsusi* | 103.92 | 27.72 | 32.46 | 0.71 |
| RH250 | ML17116 | *R. bachii* | *Tsutsusi* | 109.95 | 25.60 | 42.84 | 0.68 |
| RH292 | MFR019 | *R. bachii* | *Tsutsusi* | 108.81 | 28.55 | 48.32 | 0.66 |
| RH293 | CXN201926 | *R. bachii* | *Tsutsusi* | 107.11 | 28.99 | 37.12 | 0.65 |
| RH297 | MYJ015 | *R. cavaleriei* | *Tsutsusi* | 108.24 | 25.52 | 24.17 | 0.62 |
| RH177 | ML17104 | *R. championae* | *Tsutsusi* | 110.19 | 24.15 | 43.11 | 0.73 |
| RH227 | MSC1709 | *R. chunii* | *Tsutsusi* | 109.95 | 25.61 | 25.19 | 0.72 |
| RH282 | MYJ030 | *R. florulentum* | *Tsutsusi* | 114.16 | 26.56 | 31.67 | 0.67 |
| RH212 | 2018R007 | *R. hancockii* | *Tsutsusi* | 102.73 | 25.13 | 45.46 | 0.63 |
| RH298 | MYJ006 | *R. henryi* | *Tsutsusi* | 110.19 | 24.15 | 30.09 | 0.63 |
| RH291 | MFR013 | *R. hongkongense* | *Tsutsusi* | 114.07 | 26.43 | 44.66 | 0.66 |
| RH229 | MSC1738 | *R. huadingense* | *Tsutsusi* | 121.09 | 29.25 | 41.97 | 0.71 |
| RH235 | LZQ_2015_08 | *R. kwangtungense* | *Tsutsusi* | 112.92 | 24.94 | 43.49 | 0.72 |
| RH271 | Ljq_Zl_005 | *R. latoucheae* | *Tsutsusi* | 106.72 | 26.62 | 32.13 | 0.72 |
| RH286 | MYJ017 | *R. latoucheae* | *Tsutsusi* | 111.01 | 26.38 | 39.19 | 0.63 |
| RH289 | ML2019243 | *R. leptothrium* | *Tsutsusi* | 99.27 | 27.28 | 45.94 | 0.65 |
| RH283 | ML201914 | *R. malipoense* | *Tsutsusi* | 104.82 | 23.21 | 32.59 | 0.65 |
| RH249 | ML17100 | *R. mariae* | *Tsutsusi* | 110.11 | 23.97 | 45.13 | 0.72 |
| RH208 | SWZML201747 | *R. mariesii* | *Tsutsusi* | 108.68 | 31.60 | 37.57 | 0.69 |
| RH285 | SWZML201751 | *R. mariesii* | *Tsutsusi* | 108.41 | 31.61 | 39.93 | 0.64 |
| RH280 | MXXLJQ002 | *R. microphyton* | *Tsutsusi* | 100.14 | 25.65 | 41.00 | 0.66 |
| RH295 | ML17080 | *R. microphyton* | *Tsutsusi* | 101.98 | 27.05 | 35.07 | 0.65 |
| RH294 | ML17102 | *R. moulmainense* | *Tsutsusi* | 110.11 | 23.97 | 20.02 | 0.62 |
| RH270 | MSC1705 | *R. rivulare* | *Tsutsusi* | 109.98 | 25.61 | 32.32 | 0.65 |
| RH296 | MYJ031 | *R. rivulare* | *Tsutsusi* | 108.24 | 25.52 | 17.52 | 0.62 |
| RH303 | Tao2019DZ | *R. schlippenbachii* | *Tsutsusi* | 124.07 | 40.37 | 39.74 | 0.64 |
| RH304 | Tao2019DZ | *R. schlippenbachii* | *Tsutsusi* | 124.07 | 40.37 | 28.03 | 0.61 |
| RH305 | Tao2019DZ | *R. schlippenbachii* | *Tsutsusi* | 124.07 | 40.37 | 30.73 | 0.62 |
| RH281 | MYJ020 | *R. seniavinii* | *Tsutsusi* | 111.01 | 26.38 | 40.84 | 0.65 |
| RH204 | SWZML201796 | *R. simsii* | *Tsutsusi* | 105.78 | 28.12 | 25.33 | 0.73 |
| RH267 | ML17123 | *R. simsii* | *Tsutsusi* | 110.46 | 25.91 | 47.39 | 0.71 |
| RH284 | ML17081 | *R. simsii* | *Tsutsusi* | 105.49 | 25.36 | 40.58 | 0.65 |
| RH022 | Liu_2017_LCG_01 | *R. stamineum* | *Tsutsusi* | 102.89 | 29.63 | 29.59 | 0.62 |
| RH023 | Liu_2017_LCG_02 | *R. stamineum* | *Tsutsusi* | 102.89 | 29.63 | 25.09 | 0.62 |
| RH991 | Liu_2017_LCG_03 | *R. stamineum* | *Tsutsusi* | 102.89 | 29.63 | 33.73 | 0.64 |
| RH287 | MYJ069 | *R. tutcherae* | *Tsutsusi* | 102.46 | 22.98 | 44.91 | 0.65 |
| RH288 | MYJ073 | *R. tutcherae* | *Tsutsusi* | 100.99 | 22.75 | 48.57 | 0.65 |

Table S9. List of polyploidy species in sequenced samples.

| Analysis ID | Species | Ploidy |
| --- | --- | --- |
| RH240 | *R. keysii* | 6X |
| RH243 | *R. xanthocodon* | 6X |
| RH062 | *R. rubiginosum* | 6X |
| RH256 | *R. rubiginosum* | 6X |
| RH187 | *R. capitatum* | 4X |
| RH087 | *R. complexum* | 4X |
| RH118 | *R. cuneatum* | 8X |
| RH070 | *R. nitidulum* | 4X |
| RH063 | *R. maddenii ssp. crassum* | 6X |
| RH117 | *R. mianningense* | 8X |
| RH161 | *R. ambiguum* | 6X |
| RH178 | *R. amesiae* | 6X |
| RH162 | *R. augustinii* | 4X |
| RH069 | *R. concinnum* | 6X |
| RH218 | *R. davidsonianum* | 6X |
| RH146 | *R. oreotrephes* | 8X |
| RH220 | *R. rigidum* | 4X |
| RH045 | *R. siderophyllum* | 6X |
| RH214 | *R. siderophyllum* | 6X |
| RH254 | *R. tatsienense* | 4X |
| RH150 | *R. yunnanense* | 6X |

**References**

1. Zhang L, Xu P, Cai Y et al. The draft genome assembly of *Rhododendron delavayi* Franch. var. *delavayi*. *Gigascience* 2017;**6**:1–11.

2. Soza VL, Lindsley D, Waalkes A et al. The *Rhododendron* genome and chromosomal organization provide insight into shared whole-genome duplications across the heath family (Ericaceae). *Genome Biol Evol* 2019;**11**:3353–71.

3. Ma H, Liu Y, Liu D et al. Chromosome-level genome assembly and population genetic analysis of a critically endangered rhododendron provide insights into its conservation. *Plant J* 2021;**107**:1533–45.

4. Zhou X, Li J, Wang H et al. The chromosome-scale genome assembly, annotation and evolution of *Rhododendron henanense* subsp. *lingbaoense*. *Mol Ecol Resour* 2022;**22**:988–1001.

5. Yang F, Nie S, Liu H et al. Chromosome-level genome assembly of a parent species of widely cultivated azaleas. *Nat Commun* 2020;**11**:5269.

6. Wang X, Gao Y, Wu X et al. High-quality evergreen azalea genome reveals tandem duplication-facilitated low-altitude adaptability and floral scent evolution. *Plant Biotechnol J* 2021;**19**:2544–60.

7. Shirasawa K, Kobayashi N, Nakatsuka A et al. Whole-genome sequencing and analysis of two azaleas, *Rhododendron ripense* and *Rhododendron kiyosumense*. *DNA Res* 2021;**28**:dsab010.
